# New Approach to Accelerated Image Annotation by Leveraging Virtual Reality and Cloud Computing

**DOI:** 10.3389/fbinf.2021.777101

**Published:** 2022-01-31

**Authors:** Corentin Guérinot, Valentin Marcon, Charlotte Godard, Thomas Blanc, Hippolyte Verdier, Guillaume Planchon, Francesca Raimondi, Nathalie Boddaert, Mariana Alonso, Kurt Sailor, Pierre-Marie Lledo, Bassam Hajj, Mohamed El Beheiry, Jean-Baptiste Masson

**Affiliations:** ^1^ Decision and Bayesian Computation, USR 3756 (C3BI/DBC) & Neuroscience Department CNRS UMR 3751, Université de Paris, Institut Pasteur, Paris, France; ^2^ Perception and Memory Unit, CNRS UMR3571, Institut Pasteur, Paris, France; ^3^ Sorbonne Université, Collège Doctoral, Paris, France; ^4^ École Doctorale Physique en Île-de-France, PSL University, Paris, France; ^5^ Laboratoire Physico-Chimie, Institut Curie, PSL Research University, CNRS UMR168, Paris, France; ^6^ Histopathology and Bio-Imaging Group, Sanofi R&D, Vitry-Sur-Seine, France; ^7^ Université de Paris, UFR de Physique, Paris, France; ^8^ Unité Médicochirurgicale de Cardiologie Congénitale et Pédiatrique, Centre de Référence des Malformations Cardiaques Congénitales Complexes M3C, Hôpital Universitaire Necker-Enfants Malades, Université de Paris, Paris, France; ^9^ Pediatric Radiology Unit, Hôpital Universitaire Necker-Enfants Malades, Université de Paris, Paris, France; ^10^ UMR-1163 Institut Imagine, Hôpital Universitaire Necker-Enfants Malades, AP-HP, Paris, France

**Keywords:** virtual reality, cloud computation, one-shot learning, inference, human-in-the-loop, MRI, CT-scan

## Abstract

Three-dimensional imaging is at the core of medical imaging and is becoming a standard in biological research. As a result, there is an increasing need to visualize, analyze and interact with data in a natural three-dimensional context. By combining stereoscopy and motion tracking, commercial virtual reality (VR) headsets provide a solution to this critical visualization challenge by allowing users to view volumetric image stacks in a highly intuitive fashion. While optimizing the visualization and interaction process in VR remains an active topic, one of the most pressing issue is how to utilize VR for annotation and analysis of data. Annotating data is often a required step for training machine learning algorithms. For example, enhancing the ability to annotate complex three-dimensional data in biological research as newly acquired data may come in limited quantities. Similarly, medical data annotation is often time-consuming and requires expert knowledge to identify structures of interest correctly. Moreover, simultaneous data analysis and visualization in VR is computationally demanding. Here, we introduce a new procedure to visualize, interact, annotate and analyze data by combining VR with cloud computing. VR is leveraged to provide natural interactions with volumetric representations of experimental imaging data. In parallel, cloud computing performs costly computations to accelerate the data annotation with minimal input required from the user. We demonstrate multiple proof-of-concept applications of our approach on volumetric fluorescent microscopy images of mouse neurons and tumor or organ annotations in medical images.

## 1 Introduction

Continuous technological advances in optical and electron microscopy have enhanced our ability to discern three-dimensional (3D) biological structures via slice-based tomography (Zheng et al., 2018; Driscoll et al., 2019; Gao et al., 2019; Hörl et al., 2019; Hoffman et al., 2020). Entire structures from organelles to whole organisms can be imaged at the nanometric resolution, allowing the exploration of complex interplay between 3D geometry and biological activity (Gao et al., 2019). Furthermore, large-scale recordings capturing entire organisms provide a new means for understanding biology at multiple spatial and temporal scales. Three-dimensional medical imaging has been accessible for many years (typically at the millimetric resolution), primarily acquired from computed tomography (CT) scans, magnetic resonance imaging (MRI), and, more recently, numerically processed ultrasound recordings. Medical image analysis is based on the specialized exploration of the slices along the principal axes of recording, i.e., the sagittal, coronal, and axial planes. These last 10 years have seen numerous machine learning-based approaches to assist and automate medical image analysis (Esteva et al., 2021).

Gaining an intuitive understanding from these complex raw data remains a challenge. Due to noise and statistical variability in the recordings, biological researchers often encounter difficulties in probing the geometry of organelles. It is also challenging in the medical imaging domain, where surgeons and clinicians lacking radiology training have difficulties in mentally transforming information in 2D image slices into a 3D representation of an organ, tumor or region of interest. In addition, natural modes of 3D visualization are missing, as most analyses rely on viewing 3D data on a computer monitor while simultaneously using a mouse to interact and extract information from the data.

Virtual reality (VR) technology has recently reemerged, in part due to low-cost consumer headsets and increasingly powerful graphics cards. The efficient integration of stereoscopy, immersion, and motion tracking in VR allows the user to visualize 3D structures in a physically realistic computer-generated environment. Interactions in this artificial environment rely on handheld VR controllers that allow physically-based actions to be performed on virtual objects.

Numerous initiatives have focused on taking advantage of this technology in the domains of education and scientific research (Dede et al., 2017; Balo et al., 2017; O’Connor et al., 2018; Johnston et al., 2018; Matthews, 2018; El Beheiry et al., 2019; Safaryan and Mehta, 2021). Recent studies have additionally highlighted the benefits of immersive viewing for handling 3D data, including efficiency and enhanced intuition relative to standard monitor-based visualization (Johnston et al., 2018; El Beheiry et al., 2019). It is worth pointing out that multiple companies have focused their efforts on developing state-of-the-art processes for high-quality image rendering. Examples of these active initiatives are found in Arivis AG and syGlass (see Table 1 in El Beheiry et al., 2020). Within the context of medical applications, initiatives have also focused on education (Djukic et al., 2013; Fertleman et al., 2018; Bouaoud et al., 2020; Shao et al., 2020; Venkatesan et al., 2021), surgery planning and diagnosis (Reitinger et al., 2006; Ong et al., 2018; Pfeiffer et al., 2018; Ayoub and Pulijala, 2019; Lee and Wong, 2019; Pinter et al., 2020; Wake et al., 2020; Boedecker et al., 2021; Chheang et al., 2021; Laas et al., 2021; Lau et al., 2021; Raimondi et al., 2021; Ruiz et al., 2021; Venkatesan et al., 2021).

Experimental three-dimensional image recordings (e.g., microscopy and medical) are typically acquired in limited quantities (Matthews, 2018). Additionally, these few acquisitions are subject to variability which make for difficult streamlining of data analysis. To address this reality we require, first, the appropriate means to visualize, interact with, and manipulate data and, second, an ability to rapidly perform quantitative assessments on these data.

The first challenge can be tackled via visualization with VR. By rendering image stacks into a VR environment, users can easily navigate and interact with their 3D data. In turn, VR enables the user to grasp an intuitive understanding of the dataset being visualized. However, multiple issues are associated with this task: 1) finding proper ways to represent diverse image stacks originating from different imaging modalities with varying signal to noise ratios, 2) providing versatile tools to explore and interact with the VR representation, and 3) finding procedures that can handle large data sets.

The second challenge is addressed by employing human-in-the-loop (Patel et al., 2019) data treatment procedures. The idea here is to couple user interactions with data analysis for extracting relevant information from the data. In the context of this work, this implies 1) defining procedures to select data within the VR environment, 2) performing the required computations for analysis without significantly impacting the VR rendering performance, and 3) allowing corrections to be performed in an iterative fashion.

In the following sections, we discuss related works involving VR software for image stack visualization. We introduce our approach, DIVA Cloud, which allows visualization and interaction in VR combined with cloud computing. Finally, we show how this approach can be effectively utilized in data annotation for microscopy and medical images.

## 2 Quick Introduction to Related Works

Affordable VR headsets, efficient graphics cards and easily accessible software development platforms (OpenXR, Unity, Unreal Engine etc.) have widened access to VR developments. These factors have promoted initiatives combining imaging techniques with VR in order to address topics in cell biology. As a result, VR applications are often forecasted to become essential components of the experimental research environment (Matthews, 2018; El Beheiry et al., 2019).

Image stack visualization in the VR environment is at the center of numerous initiatives. These include ConfocalVR (Stefani et al., 2018) and Scenery (Gunther et al., 2019), which can generate volumetric reconstructions of microscopy images. Neuroscience is a domain with a great need to visualize and manipulate data in 3D. Large images of entire nervous systems can now be acquired with optical and electron microscopy (EM). Tracing complex neuronal structures is essential as there is a link between structure, connectivity and functions of neural circuits. Some initiatives already use VR to address this (Peng et al., 2010; Usher et al., 2017). Applications at the frontiers of microscopy and neurosurgery have also been demonstrated in literature (de Mauro et al., 2009; Wang et al., 2019; Wisotzky et al., 2019).

VR interactions from multiple users on the same data have been introduced in Naviscope (Shattuck, 2018). Additionally, some projects address topic-specific challenges in microscopy, such as colocalization (Theart et al., 2017).

Companies developing software for microscopy image analysis are now adding VR compatibility for visualization and treatment. Major advances are found with Arivis AG (Dekkers et al., 2019; Conrad et al., 2020) syGlass, which include an optimized data interaction interface. Other research applications focus on biomolecule structural information visualizations and interactions (Doutreligne et al., 2014; Balo et al., 2017; Goddard et al., 2018; Cassidy et al., 2020).

Not all microscopy image analysis software involves raw, full-stack image analyses but instead a deconvolved output (Betzig et al., 2006; Manzo and Garcia-Parajo, 2015; Qi et al., 2016). It is especially the case for single-molecule microscopy, where signals from individual biomolecules are captured and processed to deduce their nanometric positions and dynamic behavior. In these cases, the microscopy image stacks are reduced to point clouds. Two recent open-source software tools have been introduced to visualize and interact with single-molecule experiments: vLUME (Spark et al., 2020) and ours, Genuage (Blanc et al., 2020). Both software offers interfaces to interact with the point clouds and to perform various forms of data analysis (measuring, counting, cropping, etc.). Other initiatives on point clouds relate to data tagging for machine learning (Berge et al., 2016; Stets et al., 2018; Ramirez et al., 2019; Wirth et al., 2019; Liu et al., 2020). Mixed applications can be found involving astronomy, such as Gaia Sky (Sellés, 2013). General visualization and interaction software include PointCloud XR and developments centered on compression to ensure visualization in VR and Augmented Reality (AR) (Pavez et al., 2018).

In medicine, VR has found applications in surgery-specific topics, notably education. While VR may be useful for radiology (Uppot et al., 2019; Elsayed et al., 2020), radiologists are trained to perform 3D mental reconstructions of medical images, limiting their interest in immersive visualization modalities. Craniofacial trauma education (Bouaoud et al., 2020), neurosurgical training (Bernardo, 2017), spinal surgery (Pfandler et al., 2017), anatomy education (Uruthiralingam and Rea, 2020), orthopedic surgery (Bartlett et al., 2018; Walbron et al., 2019; Yoo et al., 2019; Lohre et al., 2020) and patient education (Dyer et al., 2018) have been demonstrated in this regard. Furthermore, clinical results hint towards uses of VR in surgical applications spanning heart diseases (Ayerbe et al., 2020; Sadeghi et al., 2020; Hattab et al., 2021; Raimondi et al., 2021), breast cancer (Tomikawa et al., 2010; Laas et al., 2021), liver surgery (Reitinger et al., 2006; Quero et al., 2019; Golse et al., 2020; Lang and Huber, 2020; Boedecker et al., 2021), pediatric surgery (Wang et al., 2012; Ruiz et al., 2021; Salvatore et al., 2021) and orthopaedic surgery (Bartlett et al., 2018; Yoo et al., 2019; Verhey et al., 2020). Multiple new companies are now investigating the potential of VR for surgical planning such as ImmersiveTouch®, PrecisionOS or SurgicalTheater.

## 3 Visualizing and Interacting With Image Stacks Without Pre-processing in VR

We recently introduced DIVA software (El Beheiry et al., 2020), a user-friendly platform that generates volumetric reconstructions from raw 3D microscopy image stacks and enables efficient visualization, analysis and quantification. The software is available at https://diva.pasteur.fr.

DIVA was developed using the Unity game engine (UnityTechnologies), and is based on what we term a lean mapper software architecture. Furthermore, the software uses the Windows-based SteamVR standard, making it compatible with most PC VR headsets, such as the HTC Vive and Oculus Rift S. DIVA renders image stacks and hyperstacks instantaneously as 3D volumes through via GPU-based volume ray-casting (Engel et al., 2004, 2017). DIVA offers a dual interface allowing the user to interact both on a standard computer monitor (i.e., desktop mode) and in an immersive artificial environment (i.e., VR mode). However, spending a significant amount of time in VR can lead to discomfort among many users. Therefore, the desktop mode allows users to set optimal visualization parameters before switching to the VR mode, which is dedicated to visually interpreting, analyzing, and navigating the data.

**FIGURE 1:**
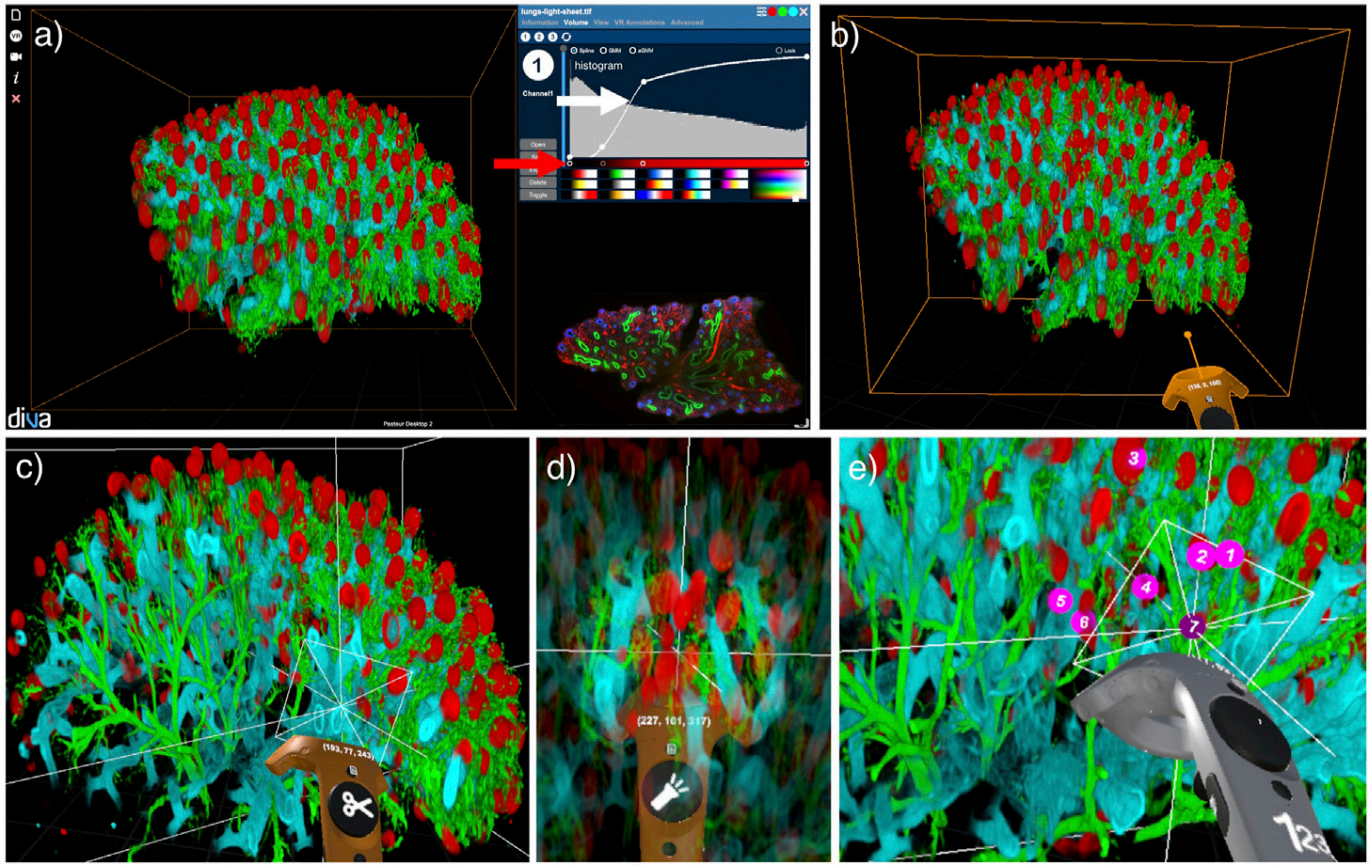
The DIVA dual interface presented on an example of a light-sheet microscopy image of a human fetus lung with pulmonary alveoli (in red), trachea (in blue) and vascular system (in green) (Belle et al., 2017). **(A)** Desktop interface with raw data in the bottom right corner and transfer function interface in the top right corner with curves for voxel opacity (white arrow) and color (red arrow). **(B)** VR interface with VR controller in orange. **(C)** Clipping tool with the VR controller to navigate inside the volume. **(D)** Flashlight tool with the VR controller to highlight a spherical area of interest. **(E)** Counter tool with the VR controller to enumerate elements of interest.

In the desktop interface, the user can modify scaling and lighting, voxel color and opacity in real-time through a user-friendly transfer function interface. This transfer function allows a simple association of color and opacity to visualized voxels based on their raw intensity values, as shown in Figure 1A. Configuration of the transfer function and interaction with the volume (rotation, translation, and scaling) can be controlled with the mouse. Transfer functions in DIVA can be saved and loaded in Javascript Object Notation (JSON) format.

In DIVA’s VR mode, the user visualizes image stacks rendered as live physical objects as a result of stereoscopy. Physical manipulation of the volume, such as grasping, rotation, or navigation, can be done with the VR controller, which acts as a 3D mouse for interaction (see Figure 1B). Hence, the data can be observed at any arbitrary angle to understand its structure in detail. To ease the navigation in complex and dense biological images, a clipping tool, presented in Figure 1C, can be activated. It dynamically removes a planar portion of the rendered volume allowing deep structures in the image to be revealed. Tagging, counting, and distance measurement tools are included for basic quantitative measurements (see Figures 1D,E). Users can extract all measurement results in a CSV file as well as through screen and movie captures.

**FIGURE 2:**
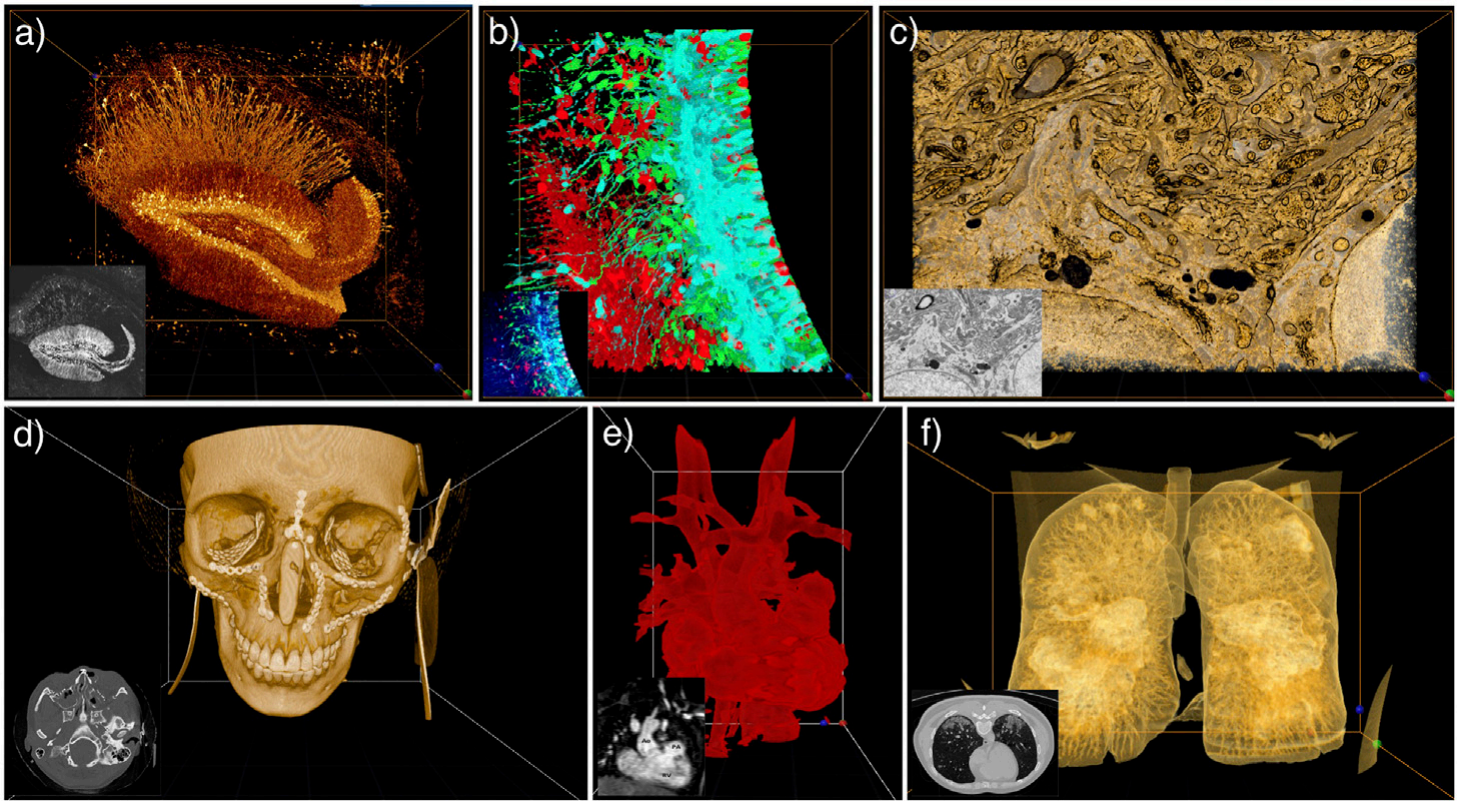
DIVA application examples on the desktop interface with their corresponding raw image in the bottom left corner. **(A–C)** TIFF image stacks of **(A)** Mouse hippocampus imaged by two-photon serial endblock imaging (SEBI, Thy-1-GFP mouse) (Sun et al., 2013). **(B)** Mouse embryonic brain slices from spinning disk microscope (Brault et al., 2016). **(C)** Focused ion beam scanning EM of components of an adult mouse neuron: Golgi apparatus and mitochondria (Gao et al., 2019). **(D–F)** DICOM images of **(D)** Post CT-scan of craniofacial fractures (Bouaoud et al., 2020). **(E)** MRI of an adult heart with ventricular D-loop and septal defect (Raimondi et al., 2021). **(F)** CT-scan of lung with COVID-19 infection (Cohen et al., 2020).

As shown in Figures 2A–C, users can utilize DIVA for various microscopy modalities with up to four different channels associated with individual transfer functions. The user can reveal structures of interest by modulating the voxel transparency and colors in the transfer function. He can also discard undesired voxels without losing information, which can happen when using segmentation techniques. For example, users can easily remove the significant background noise of EM images. DIVA can also be used to compare raw and segmented data by merging the image stacks together as TIFF files with multiple channels.

With VR, the perception of 3D structures in complex data (e.g., EM images) is enhanced, and measurements are performed quicker than in standard 2D stack viewers. Examples of advantages in 3D perception of VR are found for histological sample examination (Lobachev et al., 2021), surgery simulation or planning (Seymour, 2008; Guerriero et al., 2018; Thomsen et al., 2017; Chen et al., 2020), motion or gaze precision (Martirosov et al., 2021; Pastel et al., 2021), and 3D data labeling prior to machine learning training (Ramirez et al., 2020). Medical experts and undergraduate students have reported better visualization of 3D anatomical structures in VR using DIVA, when compared with typical 3D renderings, see Table 1 in Bouaoud et al. (2020) and Raimondi et al. (2021).

Medical images are most often stored in the Digital Imaging and Communications in Medicine (DICOM) format and analyzed through a 2D interface (i.e., a DICOM Viewer). In Figures 2D–F, we show examples of medical images visualized within DIVA. As DIVA is data agnostic, the user can experience both medical and microscopy images.

## 4 Implementation

**FIGURE 3:**
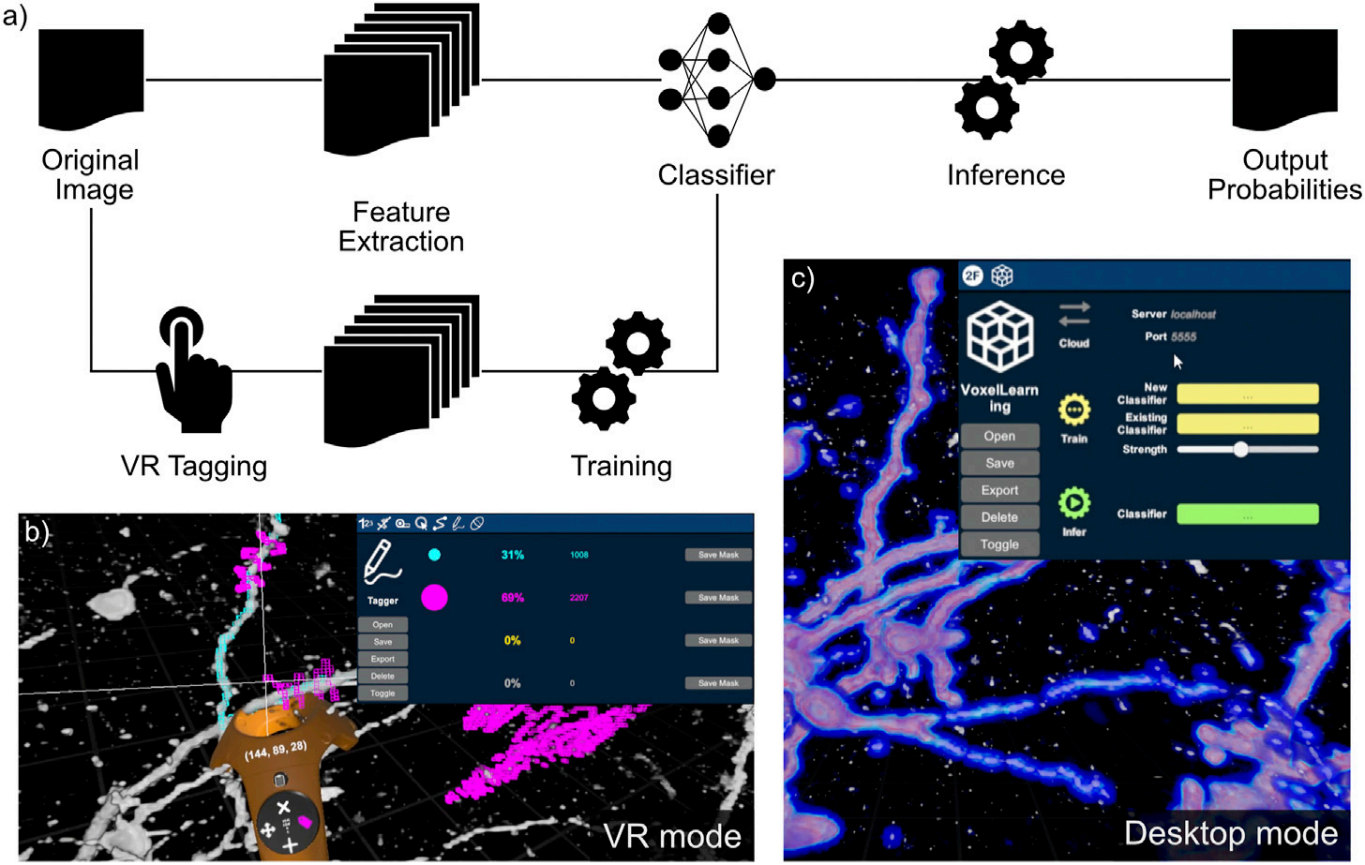
Voxel Learning and its application on a confocal image stack of mouse olfactory bulb interneurons. **(A)** Schematic of the analysis pipeline. After having set visualization parameters, the user performs the VR tagging and selects the model to be used. Training and inference steps are performed on the cloud, as indicated by the pictogram. **(B)** Data tagging step with the VR controller in orange. The positive and negative tags are colored in cyan and magenta, respectively. **(C)** Voxel Learning interface in DIVA with the output probabilities overlaid on the original image (0 corresponds to blue; 1 to red).

In this work, we seek to use the DIVA VR visualization context with 3D image analysis. We focused on the acceleration of image annotation with the objective of exploit new data without prior information or a pre-trained model. We aimed at reducing the burden of data tagging, which can require a large amount of user interaction. Our procedure consists of rapid 3D tagging in VR, simple classifier training and inference on the entire dataset with iterative corrections performed within VR. The complete procedure, Voxel Learning, is described schematically in Figure 3A and in the included Supplementary Video S1.

### 4.1 Annotation in VR and Feature Extraction

In this updated version of the DIVA software, we implemented a VR tagging functionality which allows voxel annotation i.e., associating an identifier (ID) to individual voxels. Tagged voxels appear in different colors, in a transparent wireframe mesh around the tagged voxel, allowing simultaneous visualization of the voxel and its tag. In the application shown in Figure 3B, the two colors are cyan and magenta as they highly visible in most situations. Tags can be updated or erased if necessary. The clipping plane tool (CPT) (El Beheiry et al., 2020) is also available to ensure more precision in ambiguous situations (see Figure 1C) and fluid tagging within the volume. Most importantly, the CPT allows annotating data at the frontier between different domains with geometries that do not align along the natural axis of data acquisition. Inside the VR environment, the properties of the interface and the transfer function are instrumental in accelerating the annotation process. We demonstrate the tagging procedure in Supplementary Video S2 on a medical example.

We additionally integrated the ability to calculate image features with the DIVA software. An efficient feature evaluation was implemented for each voxel, using a small subset of features (Arganda-Carreras et al., 2017). It includes a wide variety of spatial filters (Gaussian, median, mean, etc.) with different kernel sizes for gathering simple multi-scale features in the vicinity of the voxels. Features are then associated with a unique voxel ID, and the list of annotated voxels is stored. In the case of iterative tagging, the iteration number is also stored and associated with the voxel IDs.

### 4.2 Training and Inference

Our application here consists of accelerating data annotation using a simple one-shot learning procedure based on a few VR controller “strokes” performed by the user on the image stack. We follow the same principles as those used in ilastik (Berg et al., 2019), Weka (Arganda-Carreras et al., 2017) or behavior detection in larva (Masson et al., 2020), by tagging limited sets of data and stacking simple learners in order to train a collectively stronger classifier. Specifically, features are associated with tagged voxels, and learning is performed using robust classifiers in limited amounts of data. Furthermore, data tagging iterations allow the correction of anomalies in the learning to process the data.

We updated the DIVA desktop user interface to allow users to create quickly, load, and export classifiers. Basic classification approaches were used since they are known to provide robust classification on small datasets, such as Random Forest Classification (RFC), Multi-Layer Perceptron (MLP), Gradient Boosting (XGB), Support Vector Machine (SVM), and Naive Bayes (NBC) as implemented in the Scikit-learn (Pedregosa et al., 2011) Python package. In addition, hyperparameters were tuned to adapt to the problem being investigated and set to ensure rapid learning. Note that in this application, the usual problem of overfitting (Mostafa, 2012) is less present, as the goal is to annotate the data being explored and not to find a general learning scheme. Once the user has finished the annotation step, features associated to voxels are evaluated locally. They are then transferred in JSON format to the cloud, where a model is trained to classify all voxels in the 3D data stack. The models and their associated parameters are saved locally in a Pickle (Python) format. They can afterwards be loaded to perform inference on the entire dataset or, if found robust, provide initial annotations on new, previously unseen, data. The resulting inference is then broadcasted back from the cloud for local rendering in DIVA.

VR provides a significant advantage in the data annotation task by properly overlaying the classification result on the raw data in a volumetric representation. The representation of both the raw data and the annotated data provided by the classifiers helps correct errors and ensures proper annotation. We integrated a channel-based representation to DIVA in which raw data and classifier-generated data are associated with different channels allowing separate and fused views of both raw and classified data. A transfer function interface is associated for each channel. In most of our applications, the raw data was fused with the voxel probability (or log-probability) of belonging to a specific class. An example is shown in Figure 3C and in Supplementary Video S3.

Overall classifier robustness can be improved by using an ensemble of weak classifiers, whose resulting probabilities are added to the list of features before final model training. In addition, classifiers may also be iteratively updated with additional tagging rounds to correct for sub-optimal performance and false detection. Stacked learners are efficient in performing intuitive segmentation (Sommer et al., 2011). We denote here the gradient boost classifier with four weak learners as the strong learner.

### 4.3 DIVA Cloud

DIVA Cloud allows users to interact with data in VR and perform the analysis via Python scripts (see Section 6) whose calculations are performed on a remote web server. This development is motivated by the computational costs required for detailed VR volumetric rendering, leaving limited calculation bandwidth. Accordingly, to ensure fluid interactions and precise annotation dynamics, computationally costly operations are performed on the remote web server (i.e. cloud). We used Django, a Python Framework, as an application programming interface (API) provider for this project. Django features a system of data models and serializer links to a PostgreSQL database, enabling the management of jobs and file objects to track the life cycle of the learning jobs and various input and output files. This system is instrumental, as it permits performing a limited set of queries to interact with the cloud component.

The REST web service is used to provide an API with specific endpoints accessible from DIVA using HTTP methods. Celery, a task queue intermediate between the web server and the Python scripts, enables jobs to run asynchronously in a multi-threaded fashion. At the same time, the broker, Redis, allows communication with Celery.

**FIGURE 4:**
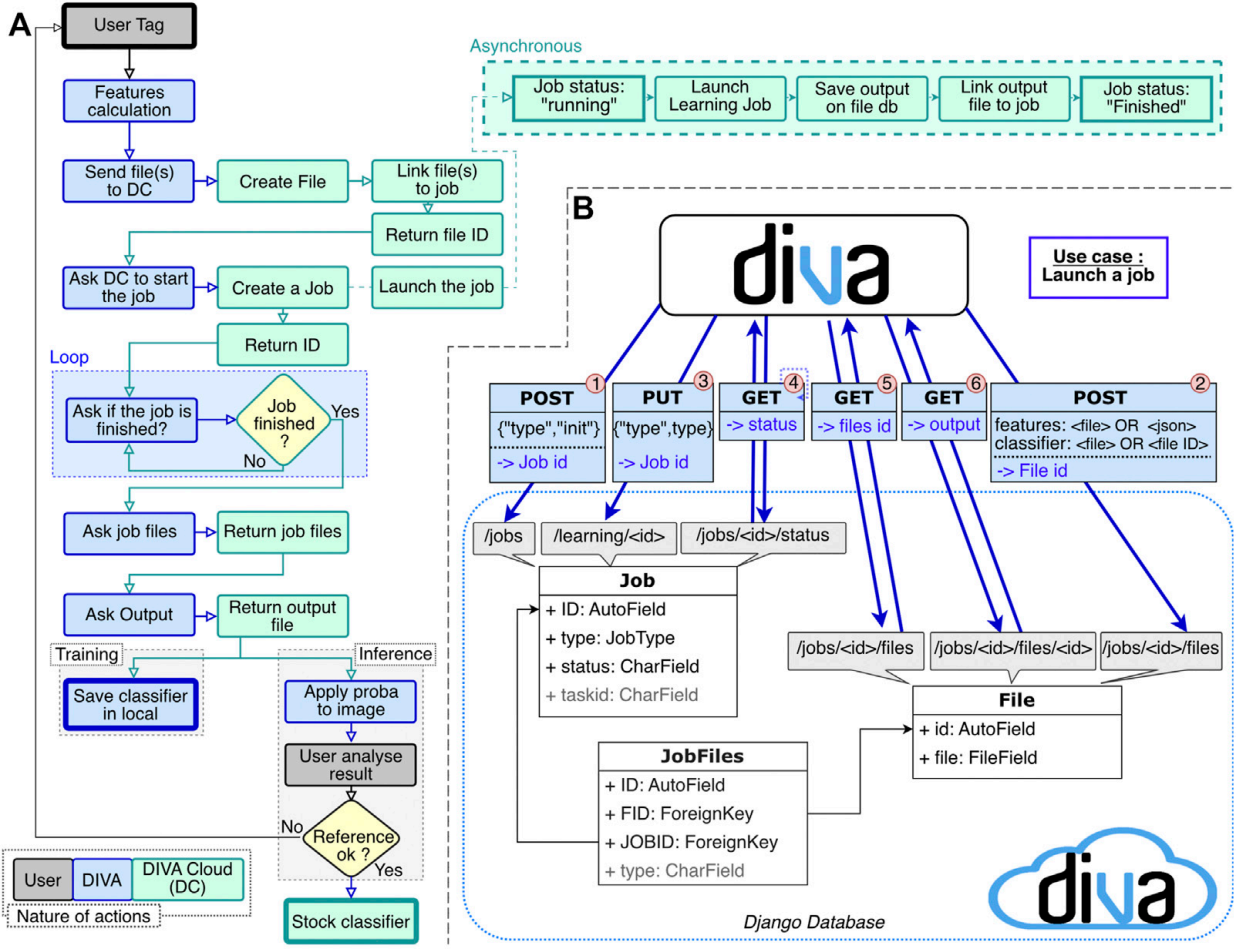
**(A)** DIVA Cloud interaction workflow through a data tagging experiment to output a classifier that is visualized in DIVA. **(B)** Interaction between DIVA and DIVA Cloud in 6 steps: 1) POST request to the/jobs endpoint. It initializes a job entry in Django. Get in return the job ID 2) POST request to the/jobs/*jobid*/file endpoint with the inputs files. It creates a file entry and returns the file ID 3) PUT request to the/learning/*jobid* endpoint with the type of learning. It launches the job on input data 4) GET request to the/jobs/*jobid*/status endpoint to know the status of the job. If the status is “running”, the status of the job is requested (Step 4 again). If the status is “done”, continue. If the status is “error”, it is managed. 5) GET request to the/jobs/*jobid*/files/endpoint to get the output list 6) GET request to the/jobs/*jobid*/files/*fileid* to download the output.

We implemented DIVA Cloud within a Docker container to ensure portability on most platforms since it does not depend on the user installation. The layers implemented in the container consist of 1) the Django webserver, 2) the Celery task queue that integrates learning scripts (with their associated packages), 3) the Redis broker, and 4) the PostgreSQL database. Note that if the user runs DIVA Cloud on a powerful computation station, the entire pipeline can be executed locally on the same computer already running DIVA. A typical interaction workflow for the DIVA Cloud application is shown in Figure 4A in the context of data tagging. A visual representation of the relation between DIVA and DIVA Cloud is shown in Figure 4B.

## 5 Results

Our analysis was performed on a Windows 10 based ×64 system with an Intel i7-7700 CPU clocked at 3.60 GhZ, with 32 GB of RAM and an NVIDIA GeForce RTX 2080 Ti graphics card. An HTC Vive headset with its controller were used through SteamVR to perform the tagging procedure in VR. This particular VR headset has a total screen resolution of 2,160 × 1,200 pixels (1,080 × 1,200 for each eye). Analysis scripts were coded in Python 3.7. The DIVA Cloud configuration was also tested on a NVIDIA DGX-1 workstation as the remote server performing the computationally challenging tasks. The frame rate of DIVA Cloud is highly dependent on the hardware configuration, image size, interface (Desktop interface in 2D or VR interface), and the user’s movements in VR. Detailed information on the frame rate regarding this study can be found in Supplementary Table S3.

### 5.1 Metrics

We showed a proof of concept of this approach on various example image stacks, including a CT-scan, MRI sequence and various microscopy images applied to neuronal specimens.

We kept track of different time measurements: the tagging step in VR never exceeded 2 min. Tagging and subsequent model training are performed on a small portion of the data. Inference duration scaled with the size of the 3D image and depended on the computational bandwidth of the cloud infrastructure.

For medical images, we used raw and annotated images in order to compare the one-shot annotation to the expert full tagging. We computed Dice coefficient and Residual Mean Square Errors (RMSE) between our inferred probabilities and the given segmentation. The goal here was to evaluate how fast annotation in VR and quick simple learning can reduce the tagging of new data to a few complementary VR strokes. Performance depends on the nature of the features extracted from the dataset. As features were designed to cover a large variety of patterns and scales, our method can see use in many additional applications and data types. Furthermore, the number and nature of the features can be extended to capture specific properties of image stacks. All measurements are available in Supplementary Tables S1 and S2.

**FIGURE 5:**
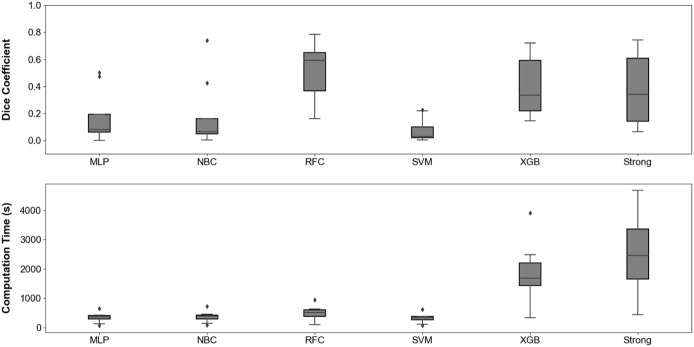
Distribution of Dice coefficient **(A)** and computation time **(B)** when applying our annotation procedure to eight different medical examples images. Corresponding raw data is available in Supplementary Table S1.

Figure 5 compares the Dice coefficient obtained with different models, along with the corresponding computing time when applied to medical examples images. The RFC and XGB, when stacked with 4 weak learners, reached highest performance with the Dice coefficient. RFC is associated to a shorter computation time, making it an appropriate candidate for efficient analysis.

### 5.2 Output Probabilities

**FIGURE 6:**
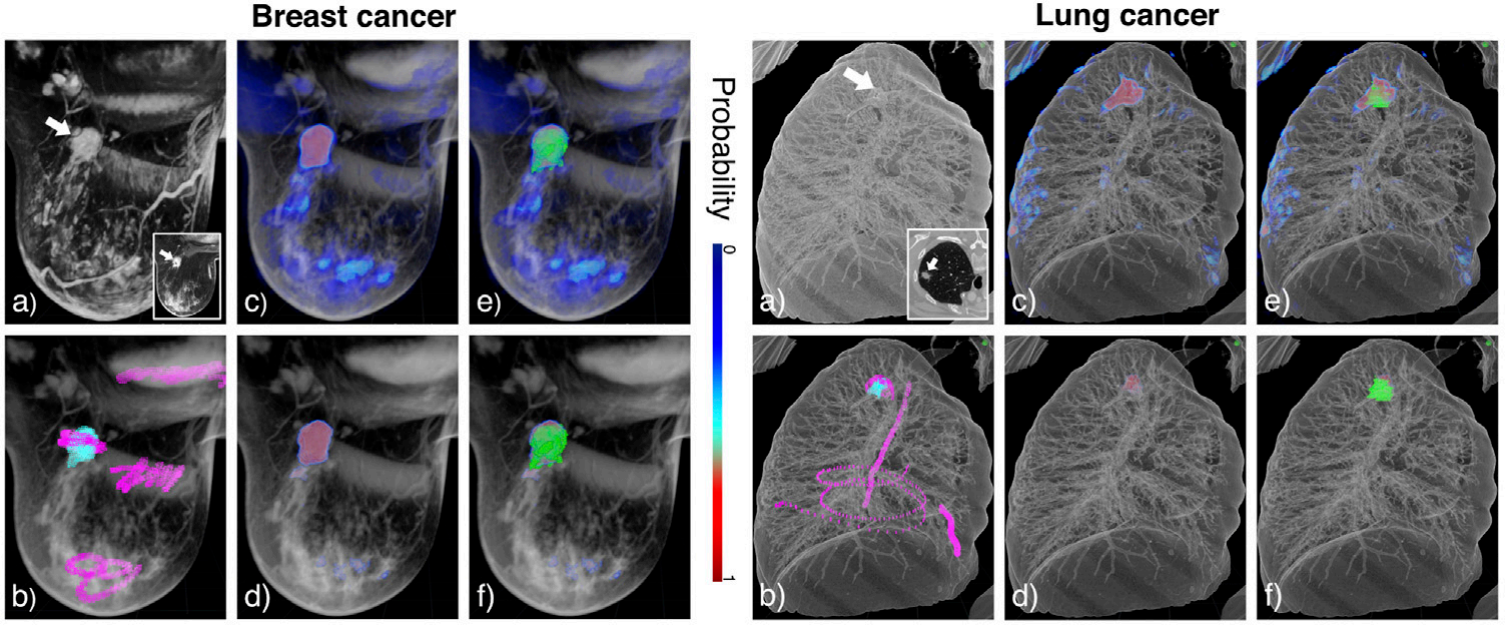
Annotation in DIVA on the breast MRI (left panel) and the lung CT-scan (right panel) and tumor (white arrow). **(A)** Raw data visualized in 3D on DIVA and as an image stack in the bottom right corner. **(B)** Overlay of the raw image in gray and tags with positive and negative tags in cyan and magenta, respectively. Tagging is performed in VR to quickly annotate which voxels belong to the structure of interest and which do not. **(C,D)** Overlay of the raw image in gray and output probabilities, respectively for the RFC and the strong learner. **(E,F)** Overlay of the raw image in gray, output probabilities, and ground truth segmentation in green for RFC and strong learner, respectively. Colorscale for probabilities is indicated between the two panels.

In order to compare the results of RFC and the strong learner, we show in Figure 6 their application to MRI images showing a patient with breast cancer and a CT-scan of a patient with lung cancer (see Supplementary Figures S1, S2 for more examples). Figure 6B exhibits the tagging step. We decided to tag the lung tumor somewhat less completely than the breast tumor in order to assess the impact of tagging exhaustiveness. Regardless, our pipeline demonstrates qualitative results, identifying the structures of interest with precision using both models (see Figures 6C,D). We note the presence of low probability inferred voxels ranging from dark to light blue for RFC, whereas the strong learner seems to classify in a more binary fashion. In this respect, RFC proves to be more prone to false positive detection. The strong learner was more stringent and, as a result, may induce more false negative errors. We observe this tendency in Figures 6E,F, as RFC predictions exceed ground truth segmentation in volume while the strong learner predictions appeared smaller.

**FIGURE 7:**
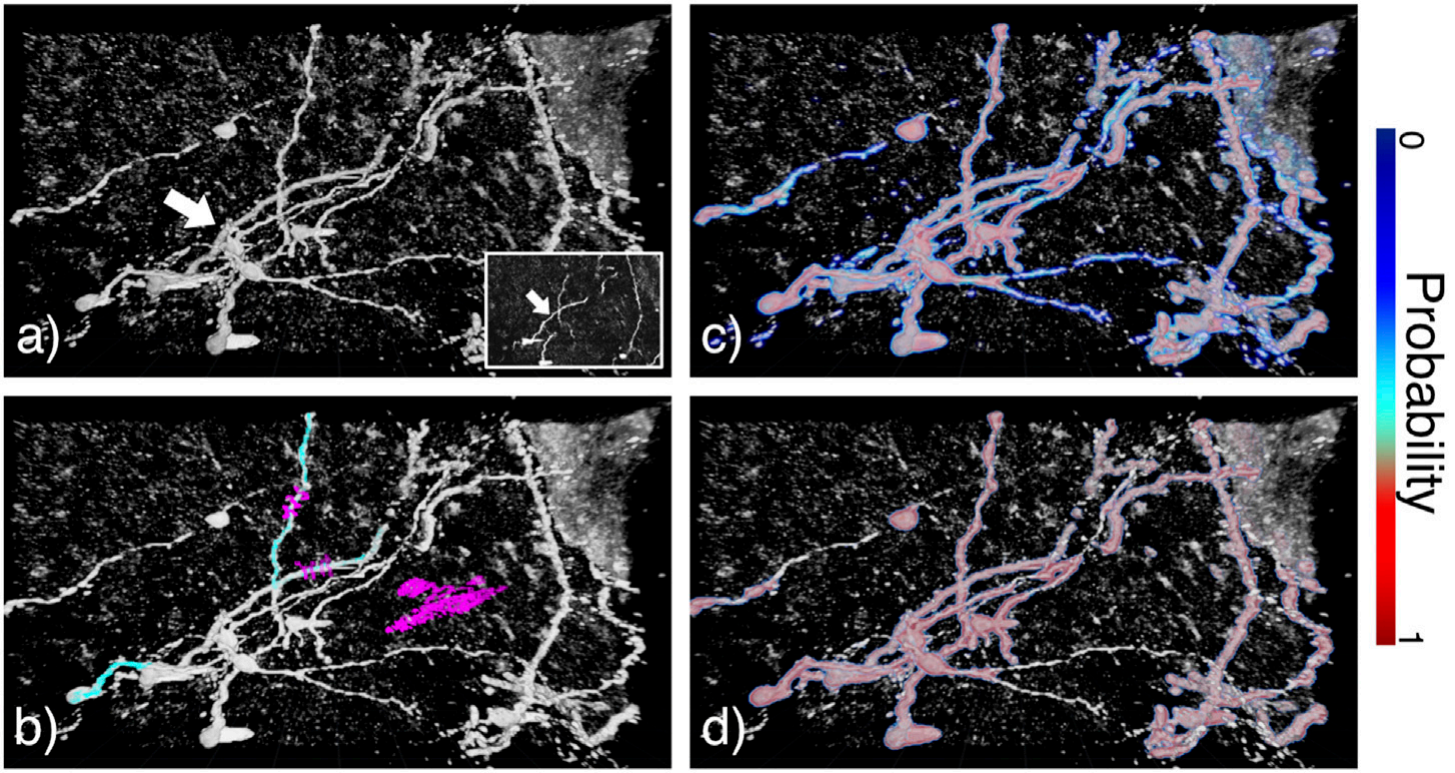
Annotation in DIVA on confocal microscopy images of mouse olfactory bulb interneurons (white arrow). **(A)** Raw data visualized in 3D on DIVA and as a z-stack in the bottom right corner. **(B)** Overlay of raw data in gray and tagging data with positive and negative tags respectively in cyan and magenta. **(C,D)** Overlay of raw data in gray and output probabilities respectively for RFC and strong learner. Colorscale for probabilities is indicated on the right of the image.

We also tested our accelerated data annotation procedure on microscopy images. Mouse olfactory bulb interneurons were imaged *via* confocal microscopy, results are shown in Figure 7. These data were considerably noisier than the CT-scan and MRI sequences that were previously utilized. The objective of this analysis was to reconstruct neuronal dendritic arbors. This was achieved by tagging two neuronal branches (see Figure 7B) and then applying our pipeline using the RFC and strong learner (see Figures 7C,D respectively). Almost every neurite and soma were classified using both learners. The RFC yielded high probabilities for inner structures and lower ones for outer structures, allowing isolating tubular structures through proper adaptation of the transfer function.

The procedure was assessed on a various range of examples: annotation of pancreas (Supplementary Figures S3, S4) and hepatic vessels (Supplementary Figures S5, S6) in CT-scans, annotation of mouse microglia in two-photon fluorescence microscopy (Supplementary Figure S7) and mouse hippocampal neurons in SEBI microscopy (Supplementary Figures S8, S9). With a total size of more than 400 million pixels, this last example confirms that our pipeline is compatible with large datasets.

### 5.3 Feature Importance

**FIGURE 8:**
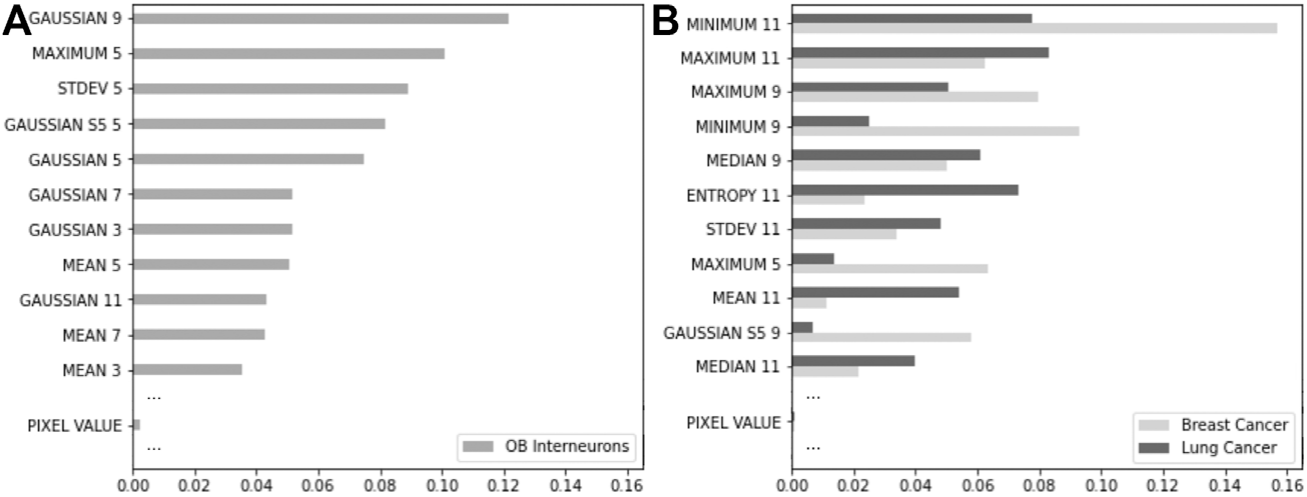
Feature importance for the RFC trained on a confocal image of the mouse olfactory bulb interneurons **(A)**, and the MRI of a breast cancer and CT-scan of a lung cancer **(B)**. The top eleven features ranked by impurity-based importance are represented, as well as the PIXEL VALUE feature.

The RFC enables a ranking of the features used during model training through a metric called impurity-based importance. The importance of a feature is calculated as the reduction in its Gini criterion. This reduction in impurity is afterward express in percentages. We show in Figure 8 the eleven most important features for the three examples previously presented, as well as the importance of *PIXEL VALUE* corresponding simply to the intensity of a given voxel (see Supplementary Figures S10, S11 for the complete features importance for the 56 features used). Interestingly, this feature, generally used in thresholding for crude denoising or segmentation, seems to have almost no importance in the final prediction.

Comparing the RFC impurity-based importance from both neuroscience microscopy and medical image types (CT-scans and MRI), we note a large variety of features that drive the final classification prediction. Indeed, for medical images, it seems that large spatial filters with 3D kernel sizes of 9–11 voxels contribute mainly to the importance, while the typical kernel size seems closer to 5 or 7 for microscopy. It can be explained by the different characteristics of the respective structures of interest (bulkier for the tumors and more tubular for the neuronal branches). While not surprising, an extended version of this procedure with larger sets of features can guide machine learning procedures that attempt to reduce the number of features used for learning.

## 6 Discussion

Virtual and augmented reality will likely play an increasingly important role in research and medical applications. More specifically, leveraging VR as part of analysis pipelines will be essential in defining algorithms and processes.

This paper focused on performing interaction and analysis in VR. We developed libraries and toolboxes allowing data annotation and analysis where computationally intensive operations are done on servers or the cloud and data fusion within the VR environment. Our approach combines several updates of the DIVA platform and a generalist interface allowing cloud computing from the VR environment. We showed proof-of-concept results on an accelerated image annotation task in which new data requires human-in-the-loop intervention to provide initial results. This provides a direct process in which limited data annotation is sufficient to train a simple statistical learning approach to classify voxels in entire image stacks.

In this work, we demonstrate the usefulness of VR in ameliorating the efficiency of data annotation tasks. The ability to grasp the volume of the data in a stereoscopic 3D viewing context and tag a small portion of it to perform an entire segmentation task is unique and promising. In contrast, the same task applied to 2D slices would require significant exploration and proves to be a tedious process.

We take advantage of the possibility of adjusting the transfer function to visualize the region of interests and their vicinity correctly. Rapidly tagging voxels not belonging to the structure of interest, by a large stroke using the VR controller (e.g., the pink streaks in Figure 6), will be instrumental in accelerating data annotation by easing learning. When compared to ground truths, the inspection of learned results is often a time-consuming task, especially when dealing with 3D data. Through its capacity to fuse different volumes in the representation, DIVA offers a suitable and time-saving environment to perform such a comparison. In turn, it allows exploring the reliability of the learning procedure and assess the quality of the ground truth itself when dealing with ambiguous data. It provides the possibility of re-annotation where anomalies are detected to feed the re-training procedure.

In this paper, we focused on the handcrafted procedure and simple ensemble learning approaches. While some of the data shown were noisy (e.g., Figure 7) or subject to artifacts (e.g., Figures 6A–F), they were relatively unambiguous. Local handcrafted features were sufficient to allow efficient semi-automated annotation. Furthermore, we limited ourselves to cases where data were completely new, and the learner would be mainly used on the explored dataset. However, complex data or the design of reusable learners may require learned features. The current pipeline can perform more complex learning. We showed (see https://github.com/DecBayComp/VoxelLearning) an example where VR annotation was directly used to capture a volume of voxels around the annotated ones and where learning was directly performed on these volumes with learned features. Finally, large-scale deep convolution-based learning can be performed by directly transmitting the full data to the cloud and using DIVA Cloud to link annotation to learning by simply exchanging voxel identifiers.

Our future initiatives will center on two topics. First, we will 1) extend DIVA and DIVA Cloud to run on AR headsets and tablets. AR involves overlaying the visual representation of the data onto the world while not immersing the user in an artificial environment. However, AR often involves representation of lesser quality than VR when used in glasses or headsets and have limited computational resources, especially when used in phones or tablets. The initiative’s core is to reduce the computational burden of full volumetric rendering to allow visualization and interaction in an AR environment. Second, we will 2) extend DIVA Cloud to allow rendering computation on the cloud and stream to the VR headset. This cloud extension will pave the way to large dataset rendering and more computationally involved rendering approaches, such as path tracing.

## Data Availability

Publicly available datasets and original contributions were analyzed in this study. This data as well as the entire pipeline can be found here: https://github.com/DecBayComp/VoxelLearning.
