# Supplementary material for: New Approach to Accelerated Image Annotation by Leveraging Virtual Reality and Cloud Computing
Source: Front Bioinform. 2022 Jan 31;1:777101. doi: 10.3389/fbinf.2021.777101 (PMC9580868; doi:10.3389/fbinf.2021.777101)
Supplement: Supplementary file 4 [file DataSheet1.pdf]

Supplementary Material

1 INTRODUCTION

This supplementary shows examples of various situations, amongst them cases where the data was complex, the tagging imperfect, or the learning not sufficient to produce acceptable results. We focused some examples on complex medical data where the current health state of the patient induced degraded images such as the one in fig S5. We also provided video captures of several processes to show how the pipeline is used, how the various interfaces work, and the advantages of VR for visualization and annotation.

The entire pipeline for *Voxel Learning* is accessible in `https://github.com/DecBayComp/VoxelLearning`. It includes an executable of the software DIVA, as well as DIVA Cloud source code and thorough instructions for its installation. In order to allow reproducibility, we provide in the directory `https://github.com/DecBayComp/VoxelLearning/materials/data_examples/` all the examples presented in the main and supplementary data. For each image, an already adjusted transfer function, tags performed in VR and trained classifiers are available in JSON format. Any user can then load and use them to recapitulate the results presented throughout this article.

2 EXAMPLES OF APPLICATION

2.1 Medical images

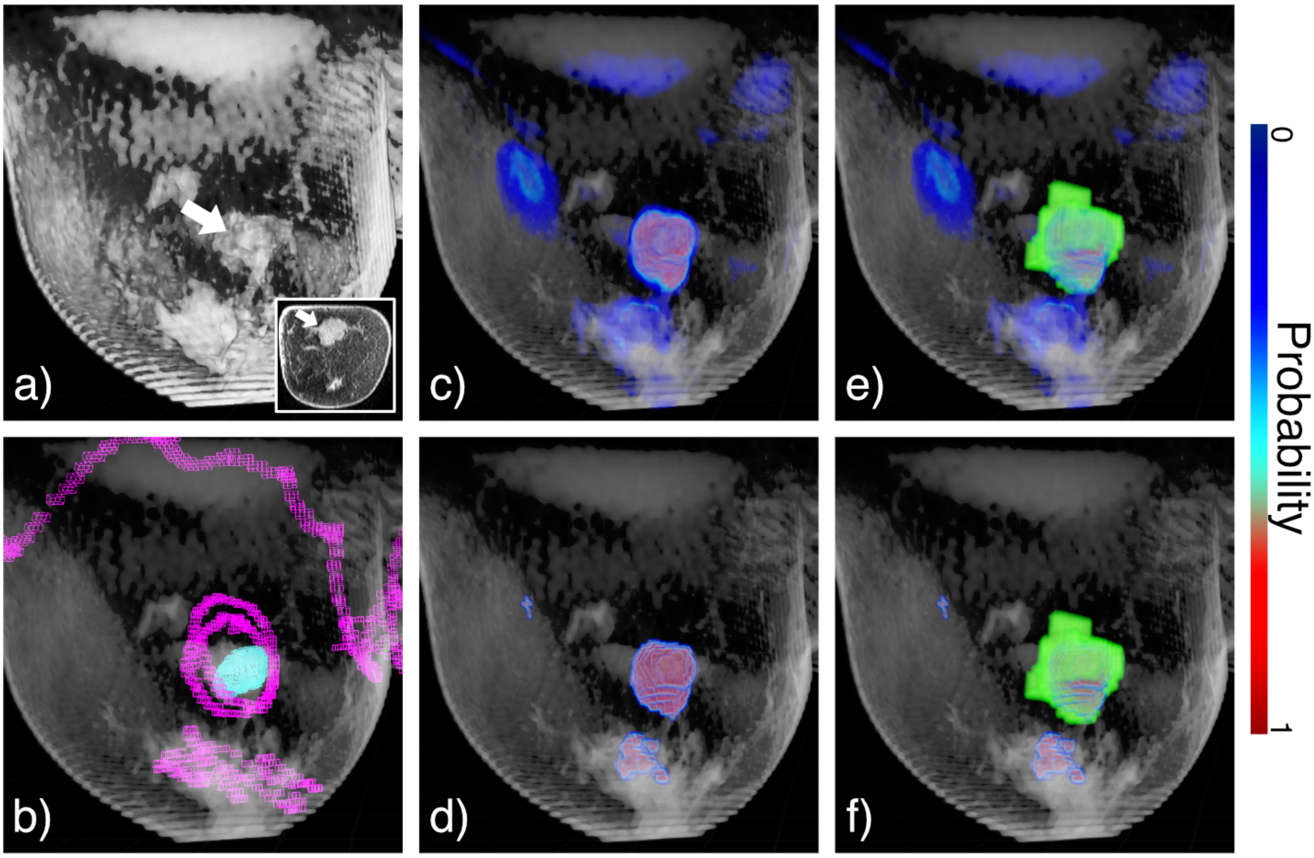

Figure S1. Annotation in DIVA on MRI of breast tumor (white arrow) from the Cancer Imaging Archive (TCIA) from the RIDER Breast MRI collection subject RIDER-1627409910.**a)** Raw data visualized in 3D on DIVA and as a z-stack in the bottom right corner. **b)** Overlay of raw data in gray and tagging data with positive and negative tags respectively in cyan and magenta. **c-d)** Overlay of raw data in gray and output probabilities respectively for RFC and *strong learner*. Colorscale for probabilities is indicated at the right.

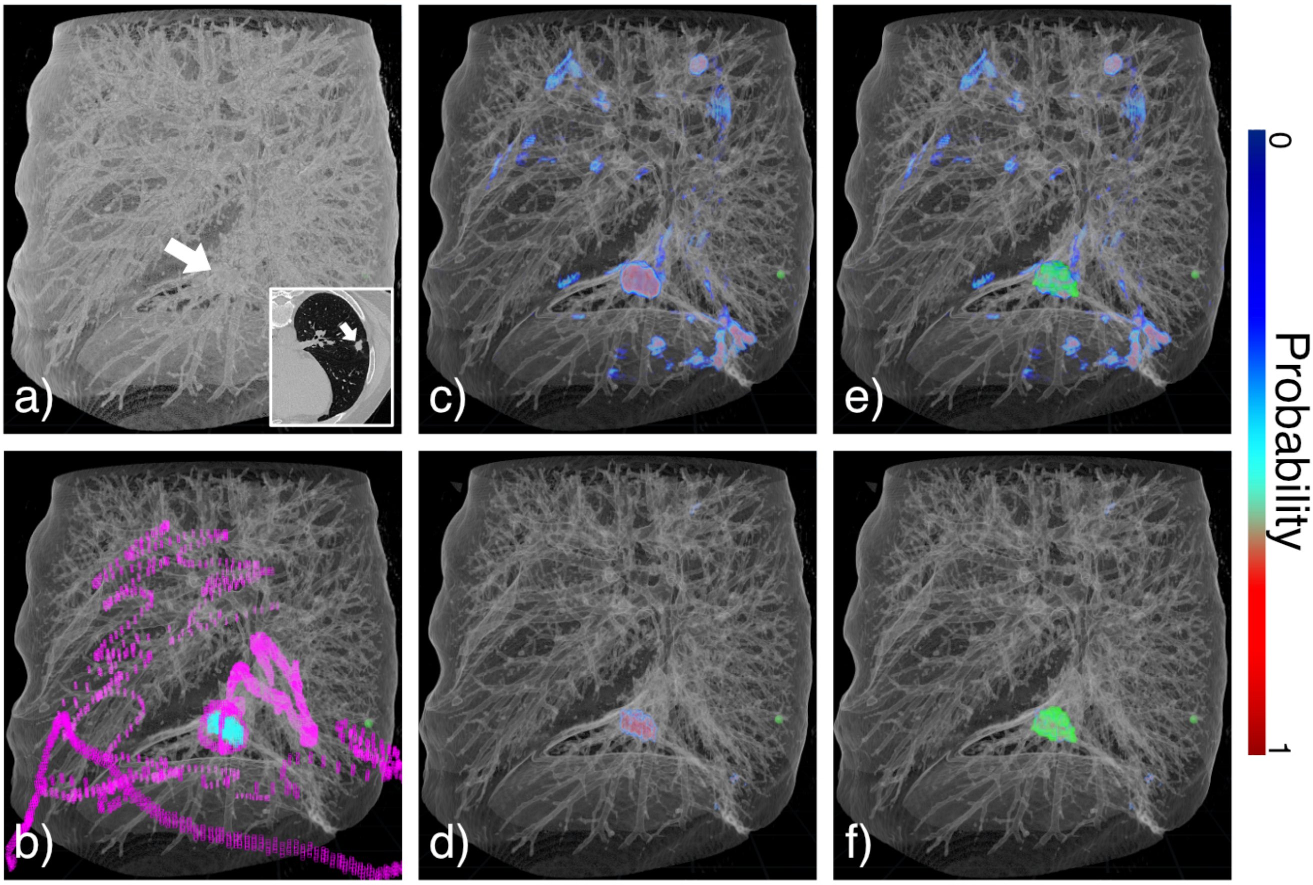

Figure S2. Annotation in DIVA on CT-scan of lung tumor (white arrow) from the Medical Segmentation Decathlon (MSD) challenge : lung_003. **b)** Overlay of raw data in gray and tagging data with positive and negative tags respectively in cyan and magenta. **c-d)** Overlay of raw data in gray and output probabilities respectively for RFC and *strong learner*. Colorscale for probabilities is indicated at the right.

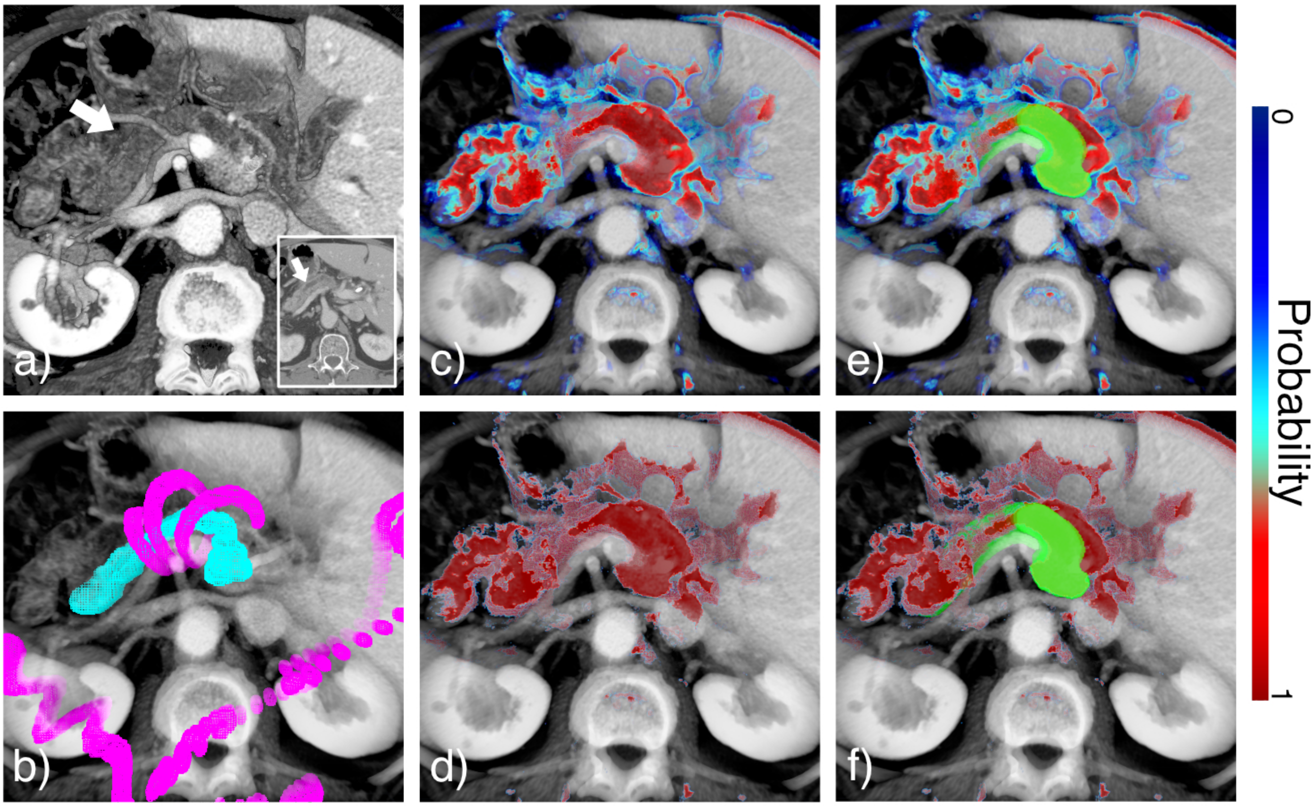

Figure S3. Annotation in DIVA on CT-scan of pancreas (white arrow) from the Medical Segmentation Decathlon (MSD) challenge : pancreas_001. **a)** Raw data visualized in 3D on DIVA and as a z-stack in the bottom right corner. **b)** Overlay of raw data in gray and tagging data with positive and negative tags respectively in cyan and magenta. **c-d)** Overlay of raw data in gray and output probabilities respectively for RFC and *strong learner*. Colorscale for probabilities is indicated at the right.

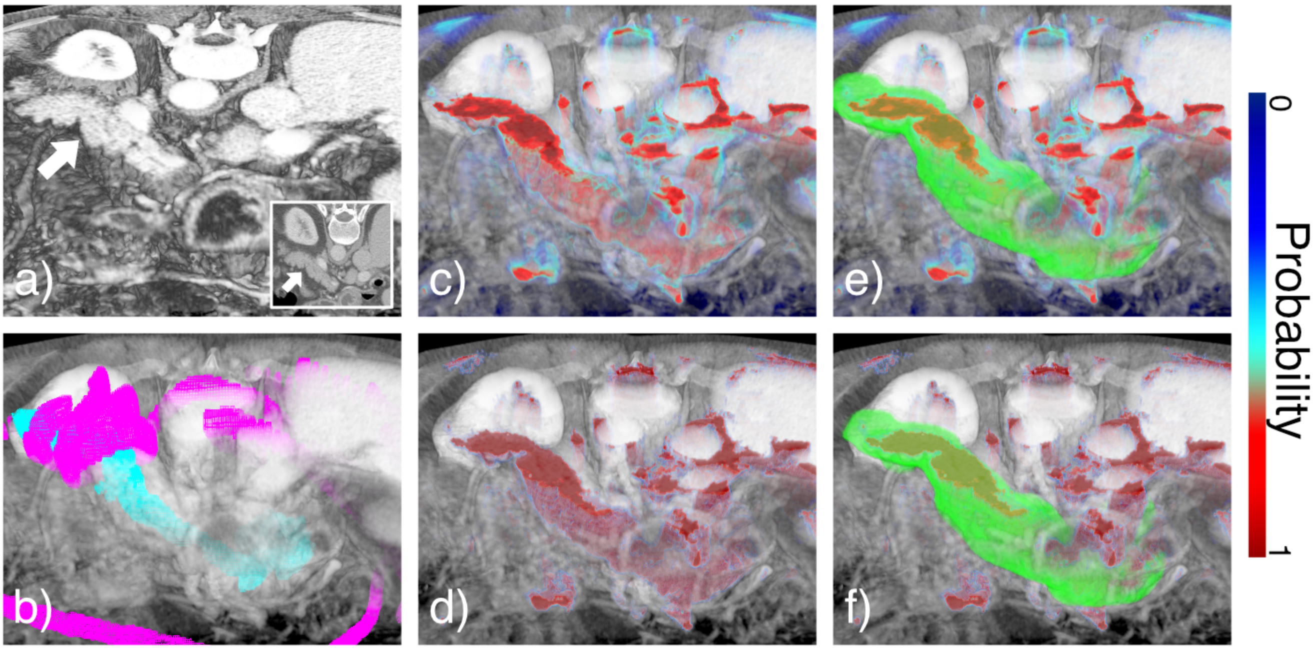

Figure S4. Annotation in DIVA on CT-scan of pancreas (white arrow) from the Medical Segmentation Decathlon (MSD) challenge : pancreas_004. **a)** Raw data visualized in 3D on DIVA and as a z-stack in the bottom right corner. **b)** Overlay of raw data in gray and tagging data with positive and negative tags respectively in cyan and magenta. **c-d)** Overlay of raw data in gray and output probabilities respectively for RFC and *strong learner*. Colorscale for probabilities is indicated at the right.

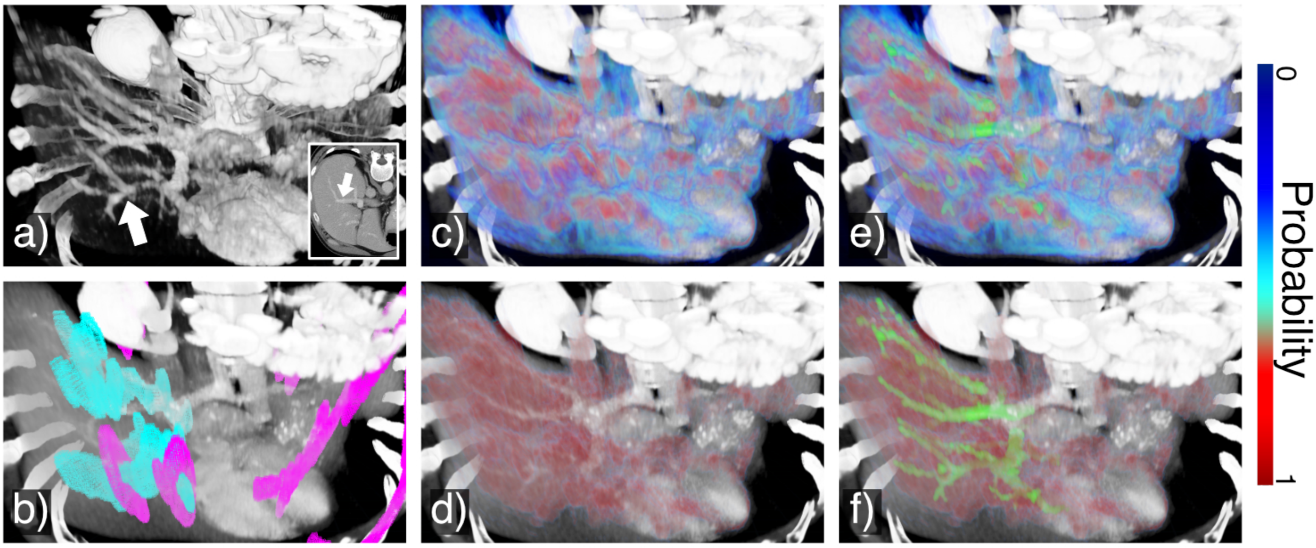

Figure S5. Annotation in DIVA on CT-scan of hepatic vessel (white arrow) from the Medical Segmentation Decathlon (MSD) challenge : hepaticvessel_001. **a)** Raw data visualized in 3D on DIVA and as a z-stack in the bottom right corner. **b)** Overlay of raw data in gray and tagging data with positive and negative tags respectively in cyan and magenta. **c-d)** Overlay of raw data in gray and output probabilities respectively for RFC and *strong learner*. Colorscale for probabilities is indicated at the right.

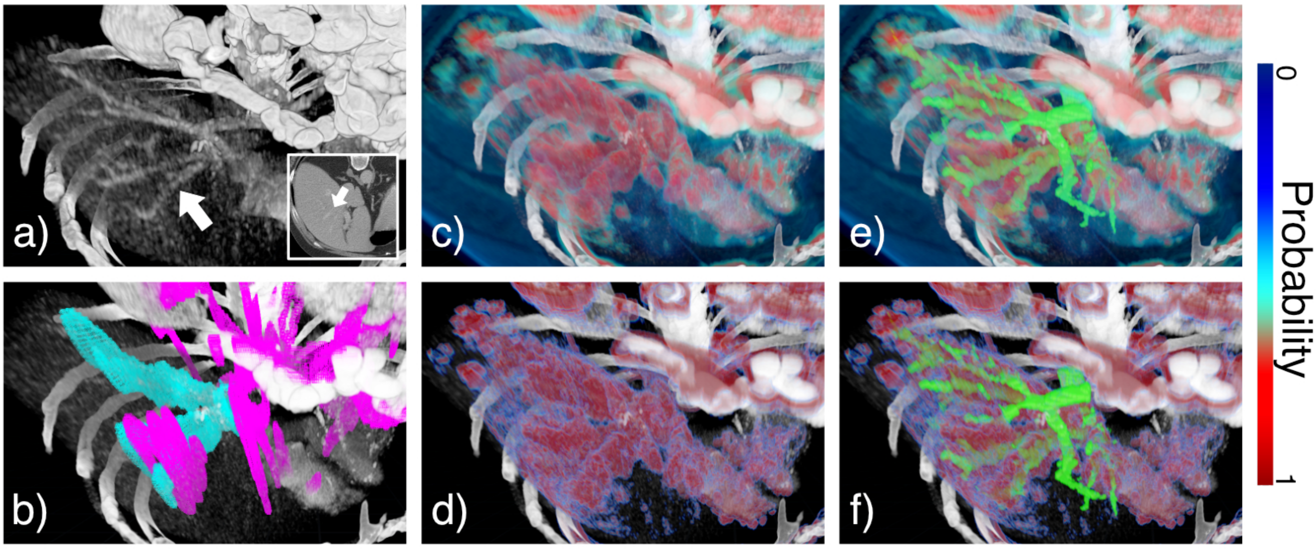

Figure S6. Annotation in DIVA on CT-scan of hepatic vessel (white arrow) from the Medical Segmentation Decathlon (MSD) challenge : hepaticvessel_002. **a)** Raw data visualized in 3D on DIVA and as a z-stack in the bottom right corner. **b)** Overlay of raw data in gray and tagging data with positive and negative tags respectively in cyan and magenta. **c-d)** Overlay of raw data in gray and output probabilities respectively for RFC and *strong learner*. Colorscale for probabilities is indicated at the right.

2.2 Microscopic images

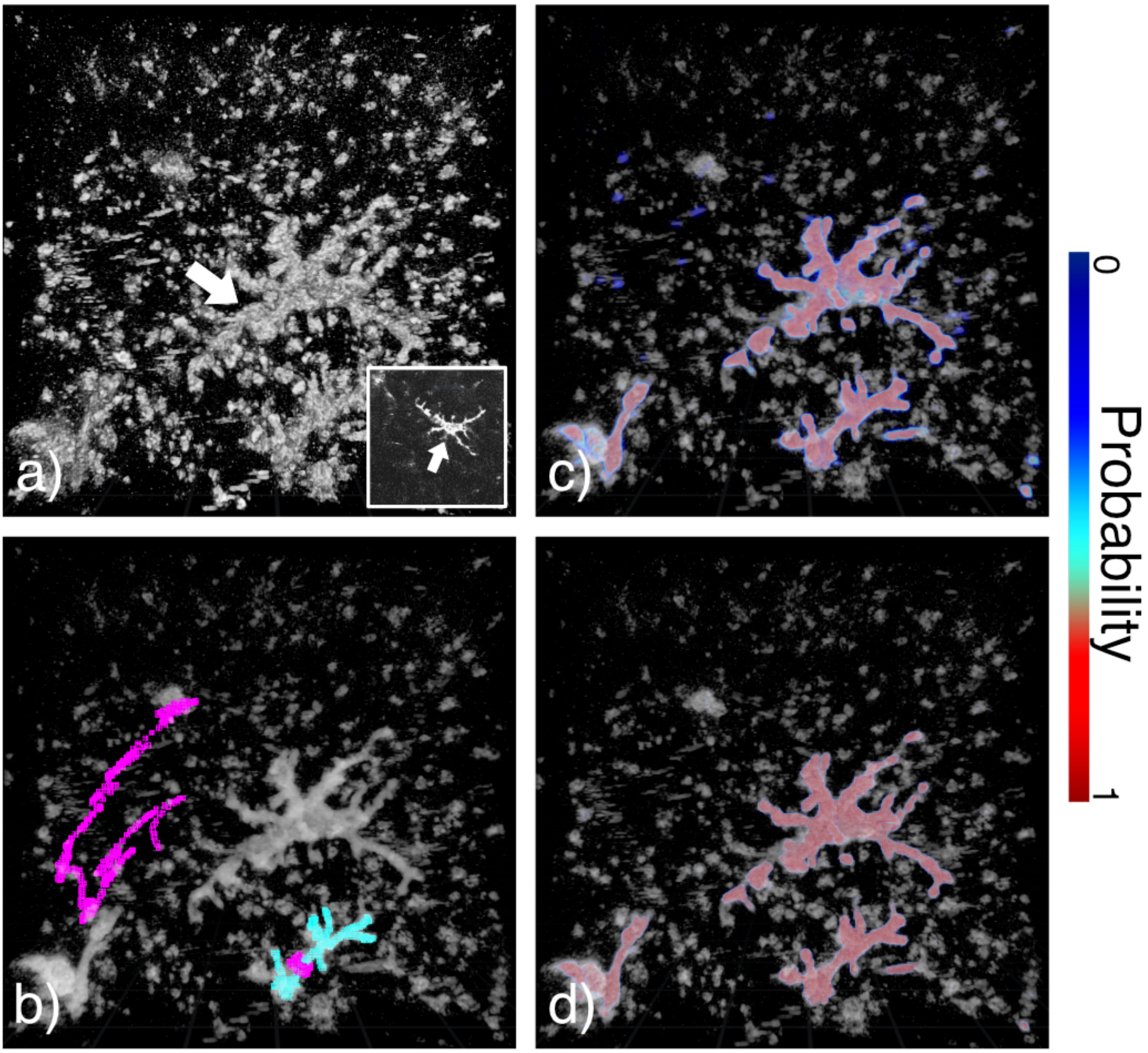

Figure S7. Annotation in DIVA on two-photon microscopy images of mouse microglia (white arrow), courtesy of Kurt Sailor (Institut Pasteur). **a)** Raw data visualized in 3D on DIVA and as a z-stack in the bottom right corner. **b)** Overlay of raw data in gray and tagging data with positive and negative tags respectively in cyan and magenta. **c-d)** Overlay of raw data in gray and output probabilities respectively for RFC and *strong learner*. Colorscale for probabilities is indicated at the right.

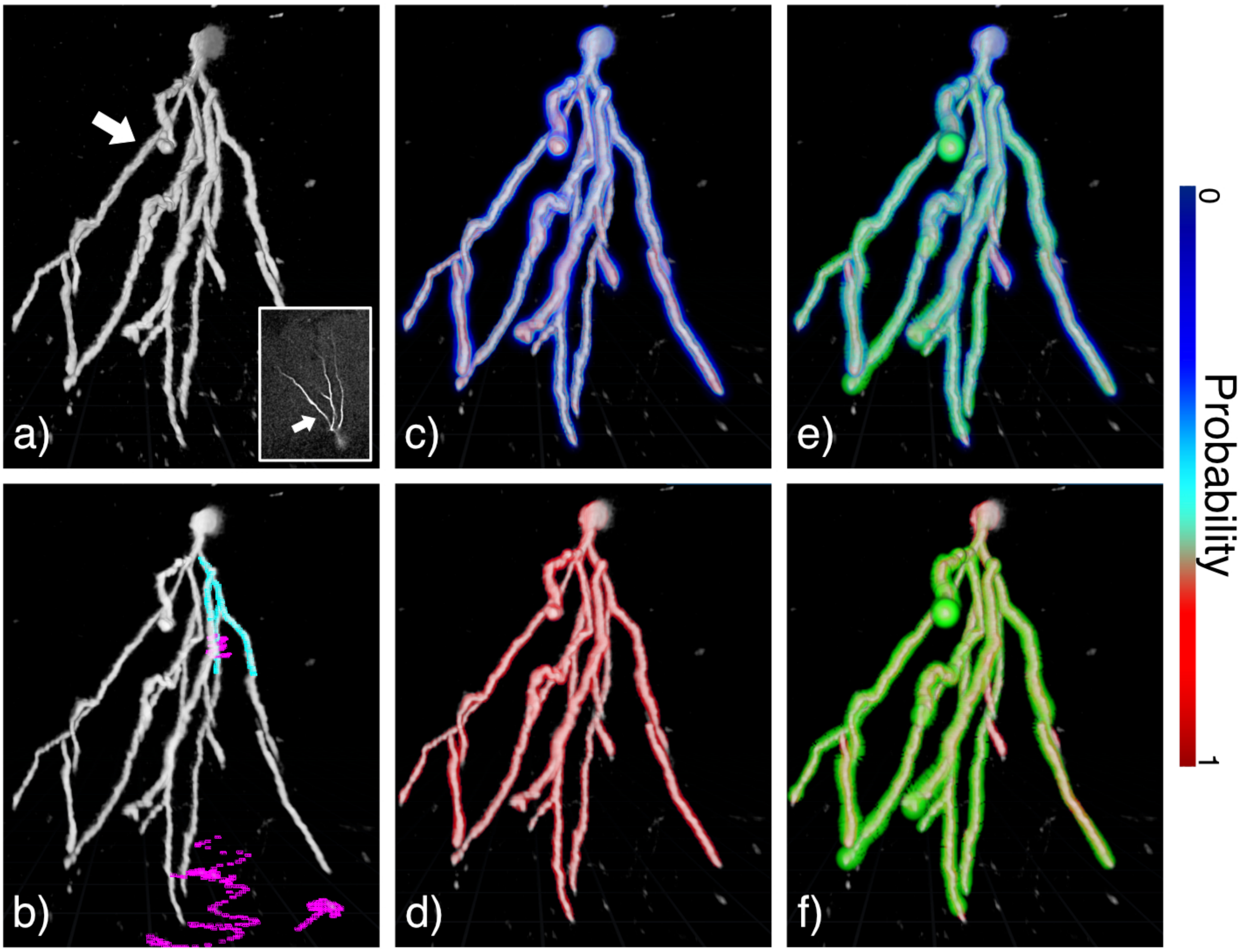

Figure S8. Annotation in DIVA on SEBI images of a mouse hippocampus neuron (white arrow), courtesy of Kurt Sailor (Institut Pasteur). **a)** Raw data visualized in 3D on DIVA and as a z-stack in the bottom right corner. **b)** Overlay of raw data in gray and tagging data with positive and negative tags respectively in cyan and magenta. **c-d)** Overlay of raw data in gray and output probabilities respectively for RFC and *strong learner*. Colorscale for probabilities is indicated at the right.

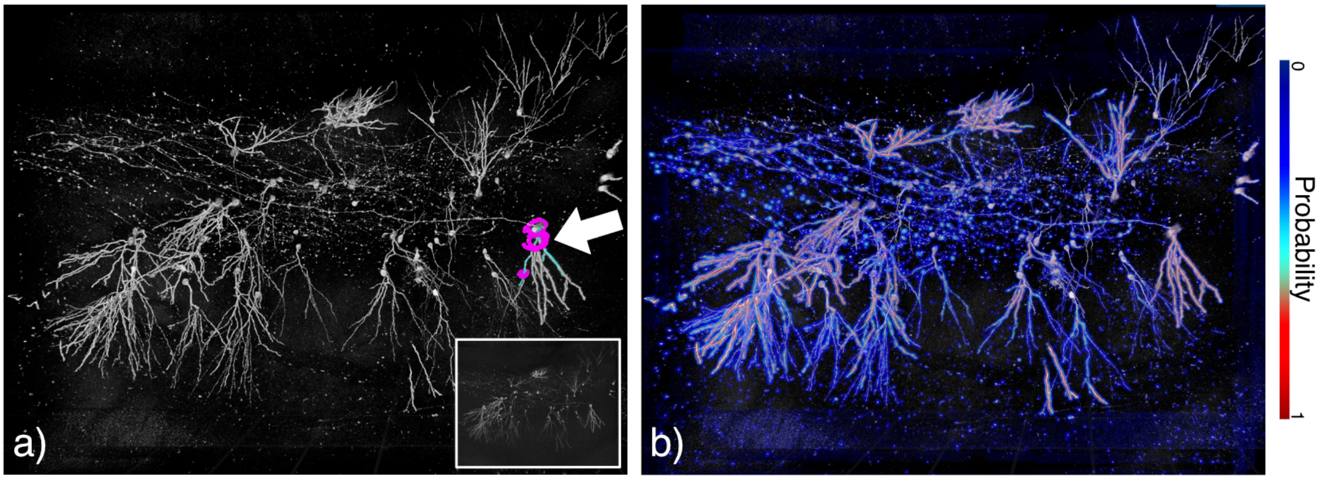

Figure S9. Annotation in DIVA on full SEBI images of mouse hippocampus neurons, courtesy of Kurt Sailor (Institut Pasteur). **a)** Raw data visualized in 3D on DIVA and as a z-stack in the bottom right corner. Overlay of raw data in gray and tagging data with positive and negative tags respectively in cyan and magenta. Tags are indicated with a white arrow. **b)** Overlay of raw data in gray and output probabilities for RFC. Colorscale for probabilities is indicated at the right.

3 FEATURES IMPORTANCE

We show here two examples of feature importance evaluation for two medical image stacks and one microscopy stack.

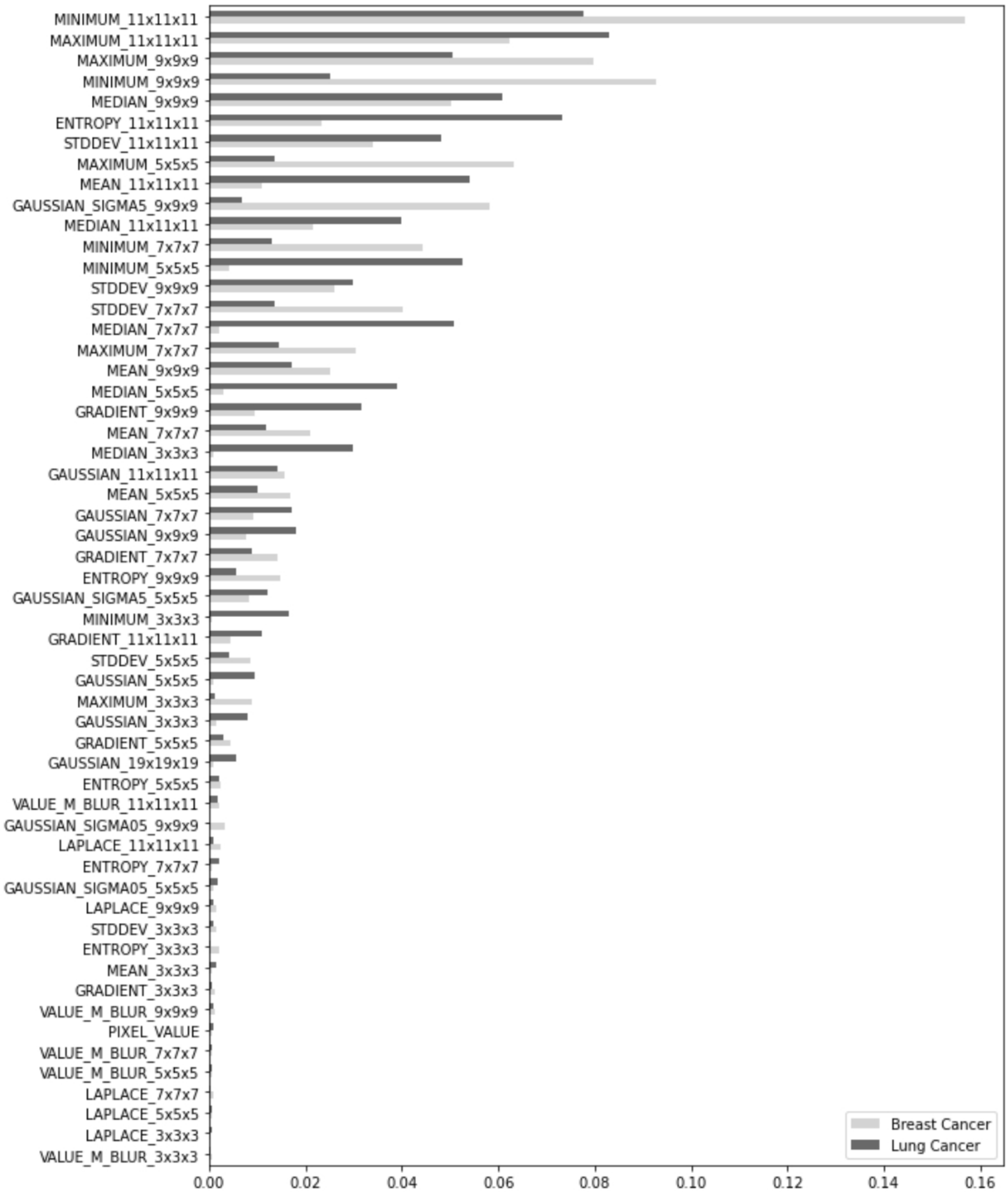

Figure S10. Features importance for RFC trained on examples of MRI of breast cancer and CT-scan of lung cancer. The comprehensive list of 56 features is here available.

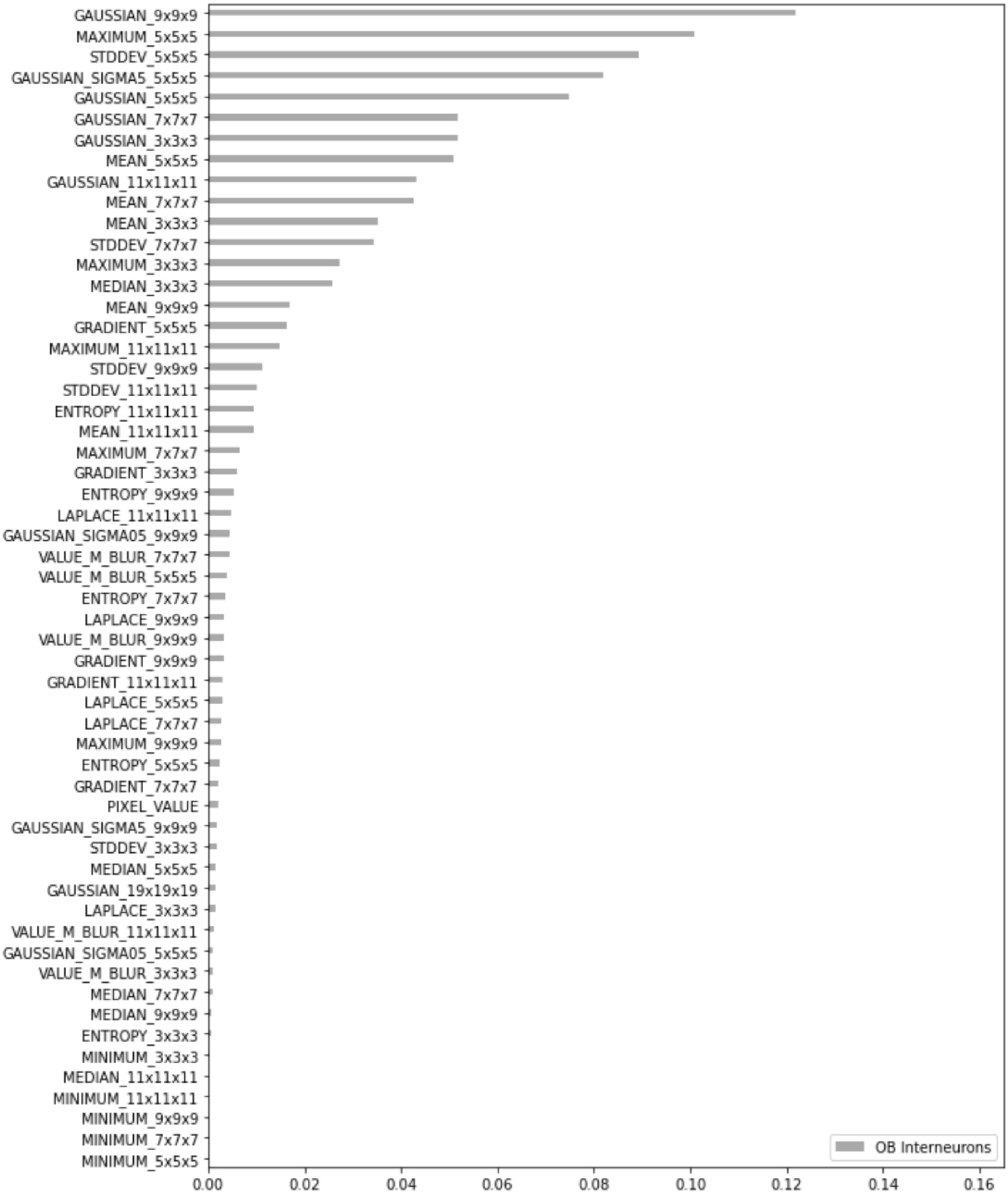

Figure S11. Features importance for RFC trained on mouse olfactory bulb (OB) interneurons example.

4 ANNOTATING IN 3D VS ANNOTATING IN 2D

We show in this section an example of a comparison between our approach to accelerated annotation and ilastik interactive segmentation (*Sommer et al., 2011*). Figure S12 shows the benefit of VR and transfer function adjustment in the visualization of complex 3D structures, here on an annotation task of hepatic vessels. In such examples, structures of interest are difficult to see in 2D sections. Volumetric reconstruction in 3D allows efficient rendering of the structure, improving thus the tagging experience. 3D motion accelerates the annotation by removing the necessity to scroll from one slide to another.

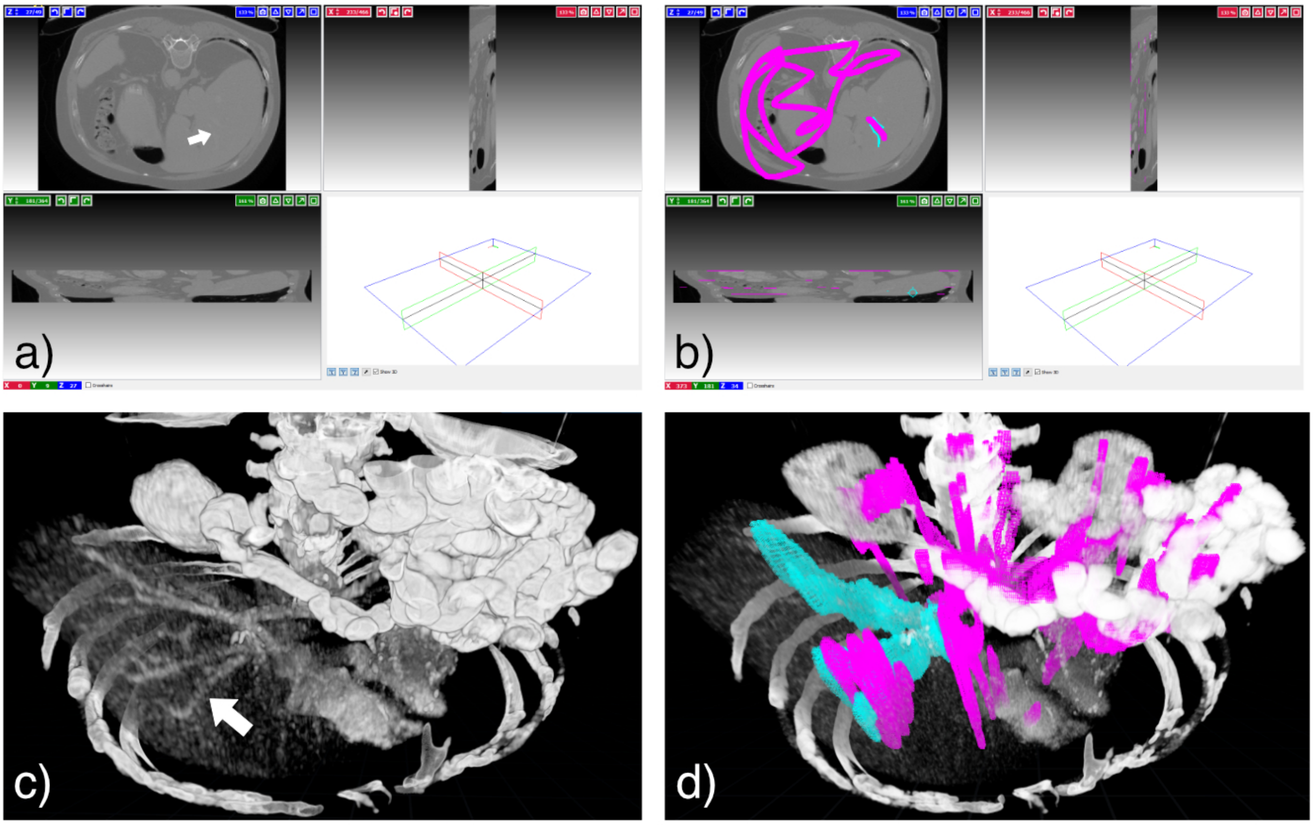

Figure S12. User experience comparison in visualization of an example CT-scan of hepatic vessel (white arrow in panels **a)** and **c)**) from the Medical Segmentation Decathlon (MSD) challenge : hepaticvessel_002. **a)** and **b)** Screenshots of ilastik interface with planar sections in the three natural axes. **c)** and **d)** Screenshots of DIVA interface, after appropriate transfer function setting. Tags are shown in **b)** and **d)** in cyan for the structure of interest, here hepatic vessels, and in magenta for the background.

We show in Figure S13 a performance comparison between ilastik and Voxel Learning applied on medical example images. Our tests were conducted in a one-shot paradigm, where the user provides limited tags: one line in the structure of interest, one line out of it. The tagging procedure should only last a few seconds. When compared to expert segmentation, we show on medical examples that our technique yields higher Dice coefficients than ilastik. Importing tags done in VR into ilastik improves its performance, while not reaching similar scores as Voxel Learning. Note that this comparison does not involve the quality

of learning as we use similar approaches as ilastik. It rather shows the expected gain from the use of VR annotation in order to provide a one-shot segmentation of the data.

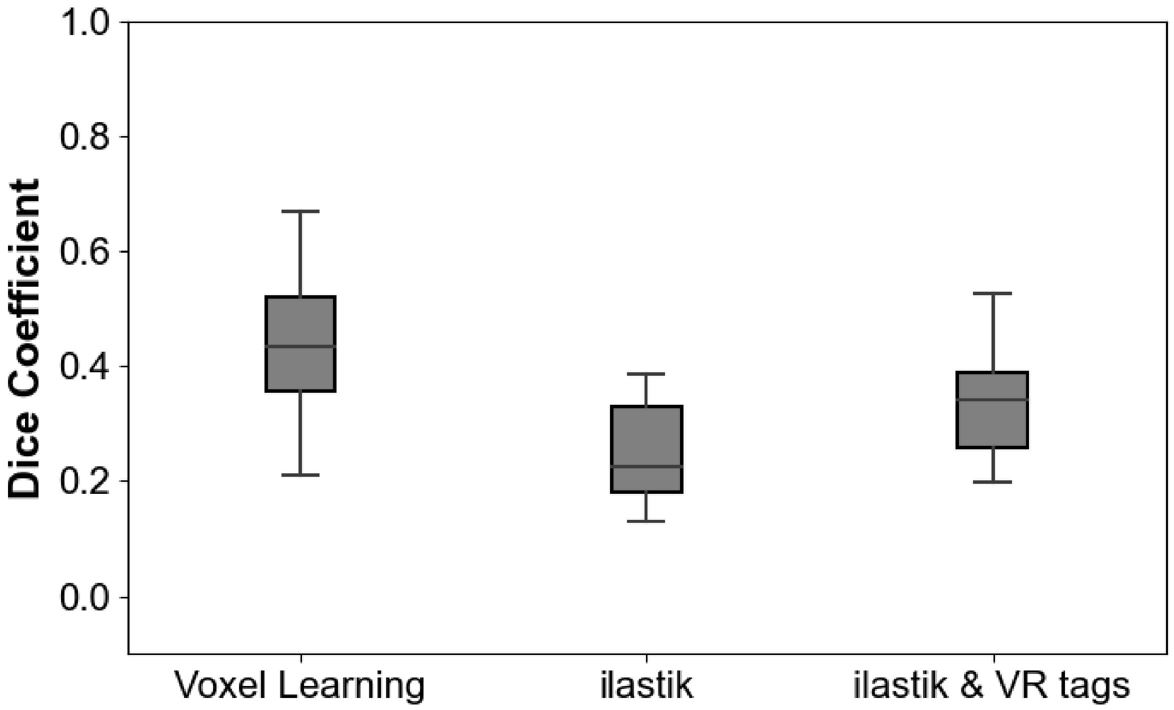

Figure S13. Performance comparison between Voxel Learning and ilastik on the 8 medical examples used throughout this article: two occurences of breast tumor MRI, and CT-scan of lung tumor, hepatic vessel and pancreas. Few tags are performed in VR for Voxel Learning, and in a 2D section in ilastik. The distribution of Dice coefficient over the different examples is shown here for Voxel Learning annotation using VR tags, and ilastik annotations respectively using 2D and VR tags.

5 A QUANTIFICATION OF EFFICIENCY IN LEARNING

In order to provide a quantitative assessment of the quality of the annotation performed within the software, we compared it to segmentation pipelines on examples of CT-scan of lung tumor. Results are shown in Figure S14. Note that such algorithms are usually trained on vast databases already annotated by experts, while our approach is based on one-shot learning, thus requiring no previous knowledge. As these results may be corrected with additional tagging, our procedure can then be iterated and performance enhanced.

a)

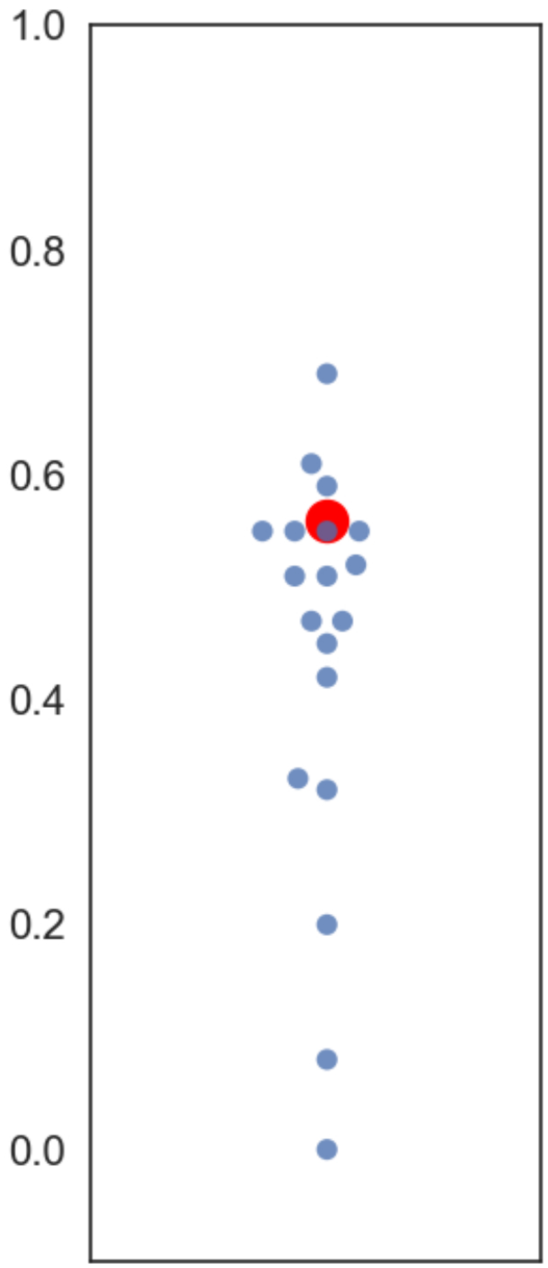

b)

Team	Dice
nnU-Net	0.69
K.A.V.athlon	0.61
CerebriuDIKU	0.59
**Voxel Learning**	**0.56**
LS Wang's Group	0.55
MIMI	0.55
Lupin	0.55
SIAT_MIDS	0.55
NVDLMED	0.52
Whale	0.51
UBIlearn	0.51
VST	0.47
LfB	0.47
A-REUMI01	0.45
BCVuniandes	0.42
BUT	0.33
Lesswire1	0.32
AI-Med	0.2
EdwardMa12593	0.08
RegionTec	0

Figure S14. Performance comparison of different segmentation pipelines on examples of CT-scan of lung tumor. We used cropped images here for practicality, and performed a single round of tagging in VR, training and inference using RFC. Dice coefficient was then assessed for 32 different examples from the MSD, and compared with the results presented in Figure 3 of *Isensee et al, 2021*. Our method obtained an average Dice coefficient of 0.56 (denoted in red), ranking 4 out of 20 segmentation algorithms (denoted in blue). A graphical representation of this comparison can be seen in **a)** with supporting data in **b)**.

6 STATISTICAL RESULTS

Modality	File Name	Original Size (number of pixels)	VR Tagging Time (s)	Tags + cyan (number of pixels)	Tags - magenta (number of pixels)	Training Features Evaluation (s)		RFC	XGB	SVM	NBC	MLP	Strong
MRI *breast_cancer*	image_01	288x288x60	30	1180	4530	26	Training Time (s)	0.58	3.27	0.18	0.06	3.10	26.60
							Inference Time (s)	131.80	679.30	68.36	92.90	73.64	832.20
							Total Time (s)	188.38	738.57	124.54	148.96	132.74	914.80
							DICE	0.78	0.72	0.22	0.74	0.47	0.74
							RMSE	0.02	0.02	0.05	0.02	0.04	0.02
	image_02	288x288x60	30	1195	3102	14	Training Time (s)	0.60	3.10	0.21	0.35	3.58	33.30
							Inference Time (s)	58.00	286.00	29.30	39.90	31.30	365.00
							Total Time (s)	102.60	333.10	73.51	84.25	78.88	442.30
							DICE	0.61	0.62	0.22	0.43	0.50	0.60
							RMSE	0.04	0.04	0.12	0.07	0.05	0.04
CT *lung_cancer*	image_01	512x512x304	60	718	15793	165	Training Time (s)	2.15	8.94	0.93	0.12	9.06	57.57
							Inference Time (s)	725.00	3677.00	393.00	506.00	417.00	4390.00
							Total Time (s)	952.15	3910.94	618.93	731.12	651.06	4672.57
							DICE	0.59	0.25	0.03	0.02	0.00	0.35
							RMSE	0.04	0.04	0.07	0.16	0.52	0.01
	image_02	512x512x288	120	556	12003	68	Training Time (s)	1.44	10.40	0.53	0.08	5.23	43.40
							Inference Time (s)	297.00	1471.00	155.00	198.00	157.00	1675.00
							Total Time (s)	486.44	1669.40	343.53	386.08	350.23	1906.40
							DICE	0.76	0.58	0.00	0.00	0.03	0.62
							RMSE	0.03	0.03	0.29	0.28	0.11	0.01
CT *hepatic_vessel*	image_01	512x512x49	85	16721	30425	60	Training Time (s)	5.66	26.06	7.76	0.28	27.95	126.60
							Inference Time (s)	486.60	2308.00	232.20	311.20	242.20	3436.00
							Total Time (s)	637.26	2479.06	384.96	456.48	415.15	3707.60
							DICE	0.16	0.15	0.03	0.08	0.10	0.07
							RMSE	0.14	0.13	0.74	0.47	0.42	0.13
	image_02	512x512x66	120	16819	62660	43	Training Time (s)	5.80	34.00	15.20	0.51	46.60	166.00
							Inference Time (s)	285.00	1464.00	144.00	189.00	153.00	1992.00
							Total Time (s)	453.80	1661.00	322.20	352.51	362.60	2321.00
							DICE	0.30	0.19	0.02	0.07	0.08	0.11
							RMSE	0.25	0.41	0.58	0.29	0.26	0.18
CT *pancreas*	image_01	512x419x51	120	43541	114081	52	Training Time (s)	28.14	71.39	40.35	0.98	30.49	353.00
							Inference Time (s)	387.00	1877.00	178.00	237.00	188.00	2711.00
							Total Time (s)	587.14	2120.39	390.35	409.98	390.49	3236.00
							DICE	0.59	0.43	0.02	0.06	0.07	0.33
							RMSE	0.14	0.15	0.75	0.47	0.43	0.15
	image_02	512x512x107	120	18883	77333	60	Training Time (s)	21.80	63.10	15.10	5.70	90.20	262.00
							Inference Time (s)	343.00	1470.00	152.00	213.00	157.00	2136.00
							Total Time (s)	544.80	1713.10	347.10	398.70	427.20	2578.00
							DICE	0.39	0.23	0.06	0.06	0.08	0.15
							RMSE	0.19	0.22	0.48	0.49	0.32	0.24

Table S1. Different measurements collected when performing data annotation with DIVA on various medical examples. For each example, the file name and the size in pixels are available. We took track as well of the tagging time in VR, the number of positively tagged voxels ("+" in cyan) and negatively tagged voxels ("-" in magenta), and the time to calculate features for the training set. For each learner, we collected the training time, the inference time and the total time for the procedure. To assess the quality of annotation, we computed the Dice coefficient and the Residual Mean Square Error (RMSE) between our results and an expert segmentation. All time measurements are expressed in seconds.

Modality	File Name	Original Size *(number of pixels)*	VR Tagging Time *(s)*	Tags + cyan *(number of pixels)*	Tags - magenta *(number of pixels)*	Training Features Evaluation *(s)*		RFC	XGB	SVM	NBC	MLP	Strong
Confocal *interneurons_OB*	image_01	315x204x39	90	1008	2207	12	Training Time *(s)*	0.60	2.70	0.40	0.20	1.70	27.00
							Inference Time *(s)*	109.00	616.00	51.60	69.70	54.00	750.00
							Total Time *(s)*	211.60	720.70	154.00	171.90	157.70	879.00
Two-photon *microglia*	image_01	255x255x63	60	1540	2914	30	Training Time *(s)*	0.70	3.60	0.40	0.28	3.80	36.00
							Inference Time *(s)*	224.00	555.00	178.00	75.00	58.00	654.00
							Total Time *(s)*	314.70	648.60	268.40	165.28	151.80	780.00
SEBI *hippocampus*	image_01	214x296x125	90	720	957	55	Training Time *(s)*	0.30	2.60	0.20	0.15	0.34	17.00
							Inference Time *(s)*	218.00	1221.00	132.00	191.00	150.00	1283.00
							Total Time *(s)*	363.30	1368.60	277.20	336.15	295.34	1445.00
	image_02	1445x1127x263	80	2253	47032	2670	Training Time *(s)*	3.98	22.30	3.66	0.33	28.80	100.70
							Inference Time *(s)*	12410.00	52794.00	7742.00	9261.00	7591.00	75626.00
							Total Time *(s)*	15163.98	55566.30	10495.66	12011.33	10369.80	78476.70

Table S2. Different measurements collected when performing data annotation with DIVA on various neuronal microscopy examples. For each example, the file name and the size in pixels are available. We took track as well of the tagging time in VR, the number of positively tagged voxels ("+" in cyan) and negatively tagged voxels ("-" in magenta), and the time to calculate features for the training set. For each learner, we collected the training time, the inference time and the total time for the procedure. All time measurements are expressed in seconds.

Modality	File Name	Image Size *(number of pixels)*	Desktop Frame Rate	VR Frame Rate
MRI *breast_cancer*	image_01	288x288x60	1100	51
	image_02	288x288x60	450	51
CT *lung_cancer*	image_01	512x512x304	100	16
	image_02	512x512x288	42	11
CT *hepatic_vessel*	image_01	512x512x49	350	50
	image_02	512x512x66	330	51
CT *pancreas*	image_01	512x419x51	500	110
	image_02	512x512x107	170	33
Confocal *interneurons_OB*	image_01	315x204x39	1050	115
Two-photon *microglia*	image_01	255x255x63	1000	51
SEBI *hippocampus*	image_01	214x296x125	330	33
	image_02	1445x1127x263	36	13

Table S3. Frame rate of the DIVA software for each image with a sampling resolution of 73 (parameter in DIVA from 0 to 100). The *Desktop Frame Rate* corresponds to the frame rate when using the Desktop interface (3D visualization of the volume on a 2D monitor). The *VR Frame Rate* corresponds to the frame rate when viewing and manipulating the volume in the virtual environment. These values are context-dependent and strongly vary upon user's actions in VR. If he navigates through the volume with the VR headset or moves too quickly, the frame rate may indeed be impacted.

7 VIDEOS

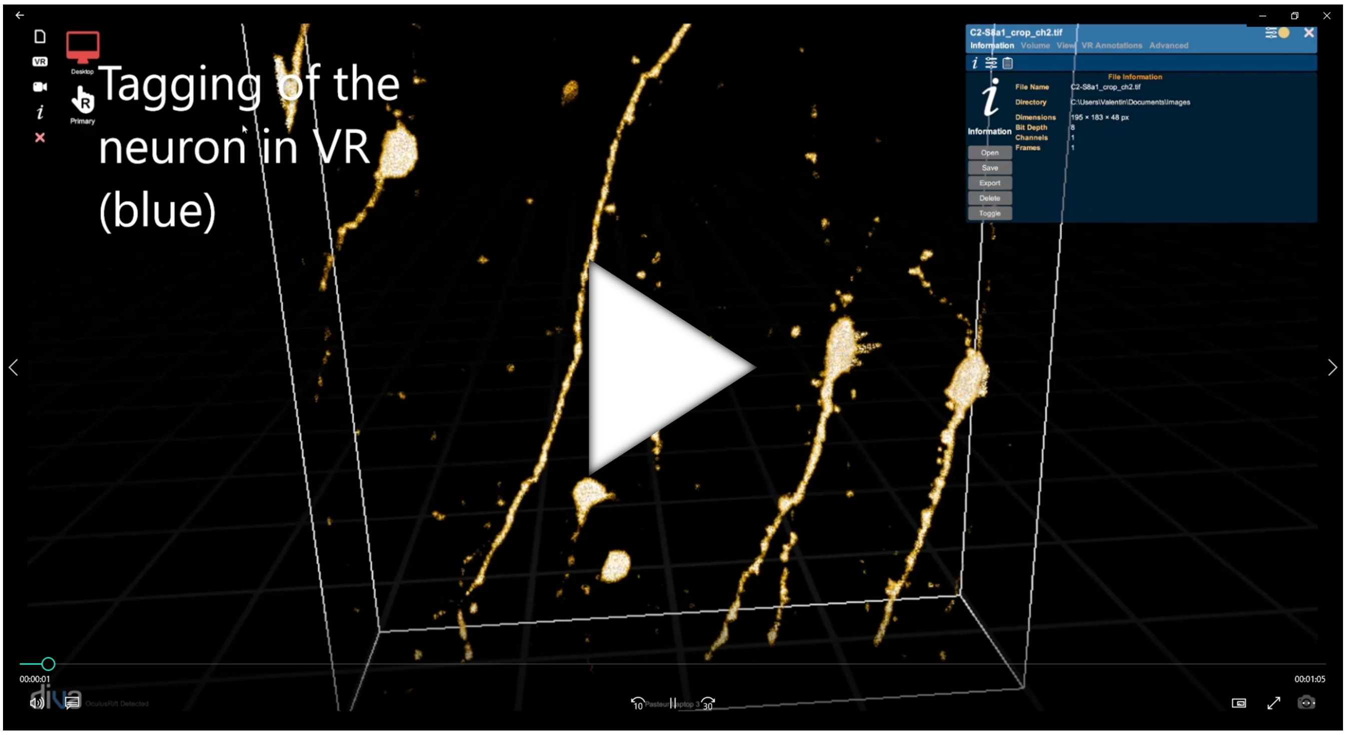

Video S1. Annotation in DIVA on confocal microscopy images of mouse olfactory bulb interneurons. The whole pipeline is comprised of successive steps. **1)** Tagging voxels in virtual reality. Overlay of raw data and tagging data with positive and negative tags respectively in cyan and magenta. **2)** Model training (here RFC) using DIVA adapted interface. **3)** Inference on the whole stack. Overlay of raw data and output probabilities for RFC. Transfer function is finally set for appropriate visualization.

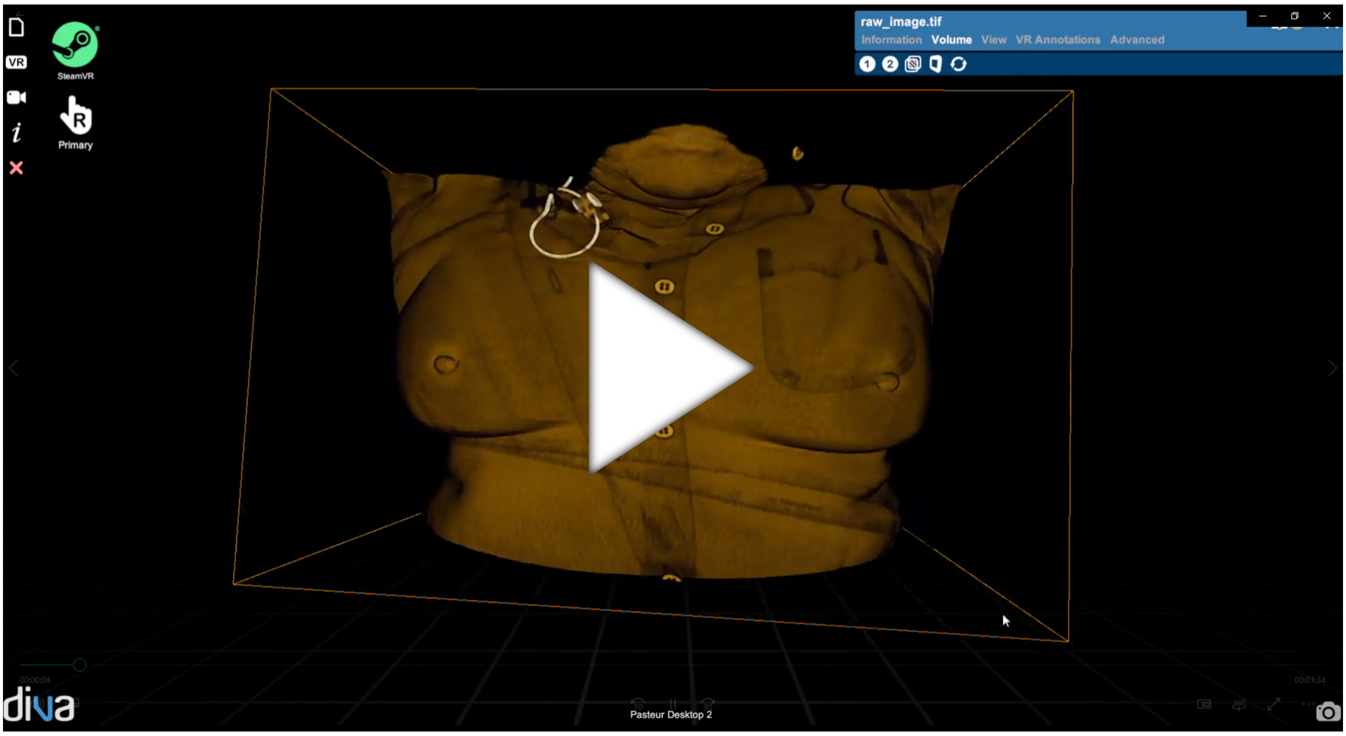

Video S2. Tagging step in DIVA on CT-scan of lung tumor from the Medical Segmentation Decathlon (MSD) challenge : lung_001. The transfer function is first set to ensure proper visualization of the tumor in desktop mode. The tagging is performed in VR using the clipping plane tool to navigate inside the volume and grasp the contour of the structure of interest. Overlay of raw data in gray and tagging data with positive and negative tags respectively in cyan and magenta.

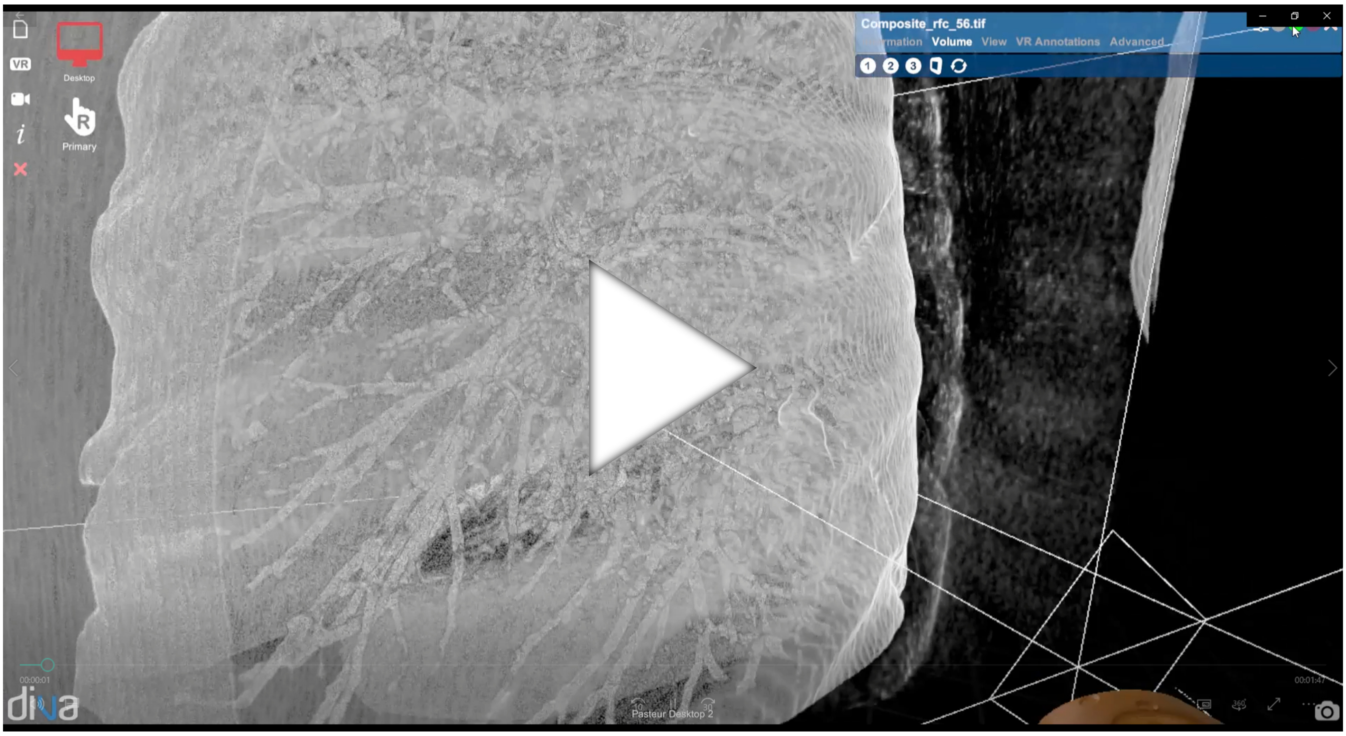

Video S3. Visualization of resulting annotation in DIVA on CT-scan of lung tumor (white arrow) from the Medical Segmentation Decathlon (MSD) challenge : lung_003. The file here has three channels, the first for the CT-scan in gray-scale, the second for the experts' segmentation in green, and the third for the inferred annotation in blue to red color-scale. These three channels are alternatively turned on and off to ensure proper visualization in VR of the structure of interest and its proposed segmentation. After using the desktop clipper, a zoom is performed on the tumor.
